# Accumulation of amyloid-β by astrocytes result in enlarged endosomes and microvesicle-induced apoptosis of neurons

**DOI:** 10.1186/s13024-016-0098-z

**Published:** 2016-05-12

**Authors:** Sofia Söllvander, Elisabeth Nikitidou, Robin Brolin, Linda Söderberg, Dag Sehlin, Lars Lannfelt, Anna Erlandsson

**Affiliations:** Department of Public Health & Caring Sciences/Molecular Geriatrics, Rudbeck Laboratory, Uppsala University, SE-751 85 Uppsala, Sweden; BioArctic Neuroscience AB, Warfvinges väg 35, SE-112 51 Stockholm, Sweden

**Keywords:** Alzheimer’s disease, Protofibrils, Glia, Phagocytosis, Degradation, Enlarged vacuole, Microvesicle

## Abstract

**Background:**

Despite the clear physical association between activated astrocytes and amyloid-β (Aβ) plaques, the importance of astrocytes and their therapeutic potential in Alzheimer’s disease remain elusive. Soluble Aβ aggregates, such as protofibrils, have been suggested to be responsible for the widespread neuronal cell death in Alzheimer’s disease, but the mechanisms behind this remain unclear. Moreover, ineffective degradation is of great interest when it comes to the development and progression of neurodegeneration. Based on our previous results that astrocytes are extremely slow in degrading phagocytosed material, we hypothesized that astrocytes may be an important player in these processes. Hence, the aim of this study was to clarify the role of astrocytes in clearance, spreading and neuronal toxicity of Aβ.

**Results:**

To examine the role of astrocytes in Aβ pathology, we added Aβ protofibrils to a co-culture system of primary neurons and glia. Our data demonstrates that astrocytes rapidly engulf large amounts of Aβ protofibrils, but then store, rather than degrade the ingested material. The incomplete digestion results in a high intracellular load of toxic, partly N-terminally truncated Aβ and severe lysosomal dysfunction. Moreover, secretion of microvesicles containing N-terminally truncated Aβ, induce apoptosis of cortical neurons.

**Conclusions:**

Taken together, our results suggest that astrocytes play a central role in the progression of Alzheimer’s disease, by accumulating and spreading toxic Aβ species.

**Electronic supplementary material:**

The online version of this article (doi:10.1186/s13024-016-0098-z) contains supplementary material, which is available to authorized users.

## Background

Knowledge about the cellular mechanisms behind initiation and spreading of Alzheimer’s disease (AD) is still very limited. Decades of research have focused on neuronal abnormalities in AD pathology, but recently more attention has been given to other cell types, including astrocytes [1]. Being the most abundant glial cell type in the nervous system, astrocytes are highly responsible for maintaining brain homeostasis [2]. Their functions include metabolic support of neurons, modification of synapse signaling, recycling of neurotransmittors, regulation of blood flow and contribution to the blood brain barrier [2, 3]. In addition, astrocytes respond to all pathological conditions through a process referred to as reactive astrogliosis, in which the astrocytes convert to an inflammatory state [4].

The amyloid cascade hypothesis suggests that amyloid-β (Aβ) mis-metabolism is the main causative event in AD, from which all other neuropathological features emanate [5]. Due to its hydrophobic nature, Aβ monomers will aggregate and form soluble aggregated species, which eventually deposit as senile plaques. A major concern with the amyloid cascade hypothesis is that the number of plaques does not correlate with the severity of dementia [6, 7]. However, results from different research groups indicate that it is the presence of soluble Aβ aggregates, such as oligomers and protofibrils, rather than the plaques that induces the widespread neuronal death [8–12]. Protofibrils have been shown to be the predominant species of soluble Aβ aggregates in both tg-ArcSwe mice and human AD brains [13, 14]. Moreover, the levels of soluble Aβ aggregates in cerebrospinal fluid (CSF) have been demonstrated to be elevated in AD patients compared to controls and to correlate with cognitive impairment [15–18].

There is compelling evidence that Aβ pathology is closely associated with inflammation and reactive astrocytes and microglia are situated tightly around the plaques [19]. The formation of a glial capsule around the Aβ deposits may protect the surrounding brain tissue from toxic Aβ species, but the astrocytes and microglia have also been shown to secrete cytokines and neurotoxic products that could induce neuronal degeneration [20]. Astrocytes effectively engulf dead cells, synapses and protein aggregates of Aβ and α-synuclein [21–27]. Interestingly, astrocytes have been shown to be more efficient than microglia in taking up Aβ, particularly during the early stages of AD [28]. The fact that reactive astrocytes with high Aβ load are frequently found in the AD brain further confirms the importance of astrocytes in Aβ clearance [29]. Compared to macrophages, that start to digest phagocytosed cell corpses directly, our group has previously demonstrated that it takes almost two weeks for astrocytes to degrade ingested, dead cells [26]. The inefficient degradation of dead cells by astrocytes is at least partly due to long lasting actin-rings surrounding the phagosomes, physically inhibiting the phagosome-lysosome fusion [30]. It is known that a large phagocytic burden could also inhibit degradation in professional phagocytes, by prolonging the time actin surrounds the phagosome [31]. Further, the astrocytes express high levels of Rab27a, a protein that reduces the lysosome acidity [30].

Since the majority of the patients with sporadic AD do not have an increased Aβ production, it has been suggested that the main cause of this form of the disease is instead insufficient lysosomal degradation [32, 33]. Moreover, it is known that patients with lysosomal storage disorders often develop neurodegenerative diseases, including AD and Parkinson’s disease [32, 34]. Ineffective degradation of Aβ may lead to spreading of AD pathology, due to secretion of Aβ-containing vesicles [35]. We hypothesized that astrocytes, due to their ineffective digestion may be an important player in these processes and the aim with the present investigation was to elucidate the role of astrocytes in Aβ_42_ protofibril clearance and toxicity. Our data demonstrates that astrocytes engulf large amounts of Aβ_42_ protofibrils that are stored in the astrocytes for a long time, rather than being degraded. This intracellular accumulation causes severe endosomal/lysosomal defects that probably reduce the degradation capacity of the astrocytes further. In addition, incomplete degradation of Aβ_42_ protofibrils results in microvesicle-induced neurotoxicity. Taken together, our results indicate that slow degradation by astrocytes may be a key process in Aβ pathology.

## Results

### Astrocytes engulf large amounts of Aβ_42_ protofibrils

**Fig. 1:**
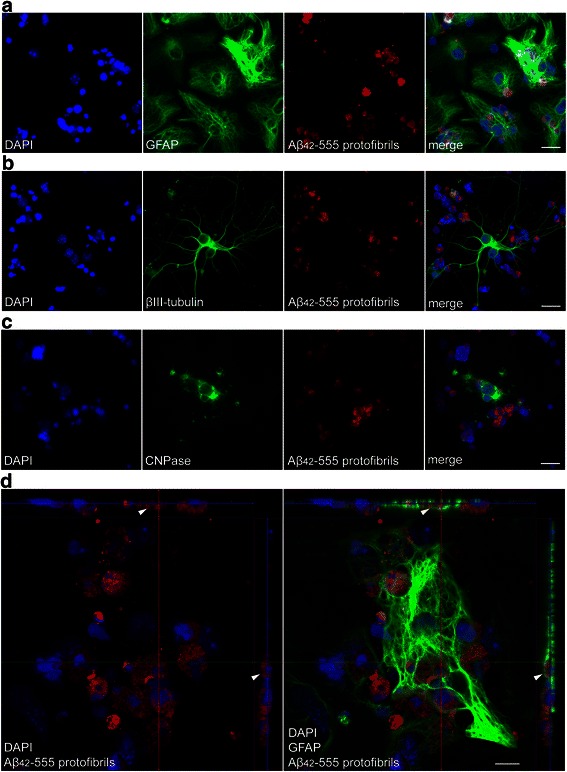
Aβ_42_ protofibril deposits are found in glial cells, but not in neurons. Immunocytochemistry of co-cultures containing astrocytes, neurons and oligodendrocytes demonstrates that astrocytes contain large deposits of Aβ_42_-555 (**a**). Neurons lack detectable Aβ_42_-555 levels (**b**), but the few oligodendrocytes in the culture also contain Aβ_42_-555 (**c**). Confocal imaging confirms intracellular localization of large Aβ_42_-555 inclusions in astrocytes (**d**). DAPI (*blue*), GFAP (*green*) and Aβ_42_-555 (*red*). Scale bars: **a**–**c** = 20 μm, **d** = 10 μm

In order to investigate the uptake of Aβ_42_ protofibrils by the major cell types in the brain, we performed experiments using co-cultures of cortical astrocytes, neurons and oligodendrocytes. The cell cultures were exposed to fluorescent HiLyte™ Fluor 555-labeled Aβ_42_ (Aβ_42_-555) protofibrils for 24 h, fixed and stained with specific antibodies to Glial Fibrillary Acidic Protein (GFAP), βIII tubulin and 2',3'-Cyclic-nucleotide 3'-phosphodiesterase (CNPase), to label astrocytes, neurons and oligodendrocytes, respectively. We found that astrocytes contained large amounts of Aβ_42_-555 (Fig. 1a and Additional file 1), while almost no Aβ_42_-555 could be detected in the neurons (Fig. 1b and Additional file 1). Interestingly, the Aβ_42_-555 inclusions in the astrocytes frequently co-localized with condensed, terminal (TdT)-mediated dUTP-biotin (TUNEL) positive cell nuclei of dead cells that had also been engulfed by the glial cells (Fig. 1a and Additional file 2). Uptake of Aβ_42_-555 protofibrils was also noted in the oligodendrocytes (Fig. 1c). However, the approximate percentage of oligodendrocytes in the cultures is very low (6 % ± 3), compared to the astrocytes (75 % ± 8) and neurons (25 % ± 8). To confirm that the Aβ_42_-555 deposits were situated inside the astrocytes we used confocal microscopy. The 3D-images clearly show that Aβ_42_-555 protofibrils were taken up and predominantly localized around condensed nuclei of dead cells inside the astrocytes (Fig. 1d and Additional file 3). It is important to remember that GFAP stains the cytoskeleton of the astrocyte, which only constitutes 15–20 % of the cell [36]. Hence, the whole astrocyte is much bigger than the GFAP staining and the Aβ_42_-555 found close to the GFAP staining is most likely also situated inside the cell (Fig. 1d and Additional file 3).

**Fig. 2:**
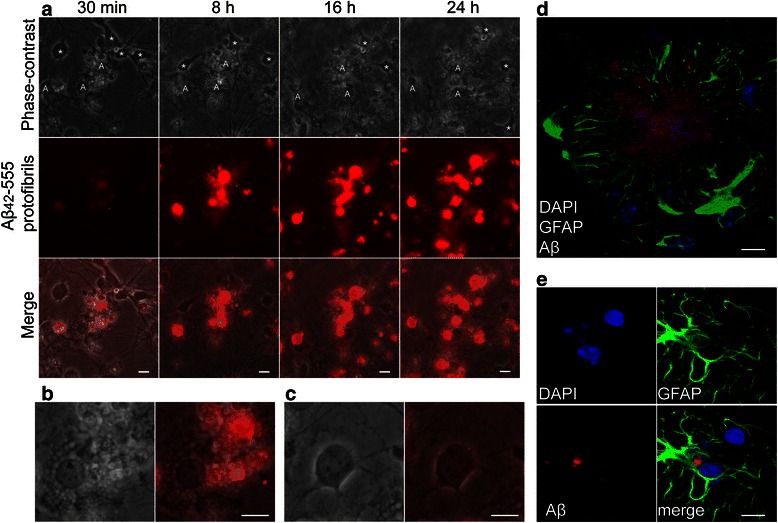
Aβ_42_ protofibrils accumulate intracellulary in astrocytes over time. Time-lapse images from 30 min to 24 h following Aβ_42_-555 protofibril administration show that astrocytes (A), but not neurons (*), accumulate Aβ_42_-555 over time (**a**). Higher magnification of a highly vascularized astrocyte with ingested Aβ_42_-555 (**b**) and a neuron with undetectable levels of intracellular Aβ_42_-555 (**c**). Immunohistochemistry of tg-ArcSwe mouse brain sections confirm that Aβ plaques are surrounded by GFAP positive astrocytes, Aβ (*red*), GFAP (*green*) and DAPI (*blue*) (**d**) and that Aβ are engulfed by astrocytes in vivo (**e**). Scale bars: **a**–**d** = 10 μm

To verify that the 555-label still bound to the Aβ after the engulfment, parallel co-cultures were treated with unlabeled Aβ_42_ protofibrils for 24 h, fixed and stained with GFAP and four different Aβ antibodies; mAb1C3 and 6E10 (both binding to the N-terminus of Aβ), mAb158 (selective for Aβ protofibrils) and a polyclonal Aβ_42_ antibody. All antibodies displayed similar staining pattern, as received with the Aβ_42_-555 protofibrils, demonstrating that the 555-signal represents the presence of Aβ (Additional file 4 A-D). To exclude that the degradation of Aβ_42_ protofibrils in astrocytes was influenced by the neurons present in the co-culture, we performed experiments with cell cultures differentiated in the presence of cilliary neurotrophic factor (CNTF). CNTF is known to drive E14 neural stem cells to generate almost exclusively astrocytes [37, 38]. The astrocytes in the CNTF-treated cultures were found to accumulate Aβ_42_ protofibrils in a similar way to those in the co-cultures (Additional file 5). To follow the engulfment of Aβ protofibrils over time we next performed time-lapse experiments, in which we recorded the cell culture during the 24 h Aβ_42_-555 protofibril exposure. Our time-lapse movies demonstrate that already after 30 min, astrocytes (identified by their phenotype of an egg “sunny side up”, large nuclei and multi-vesicular cytoplasm) had accumulated Aβ_42_-555, and the intensity of the staining constantly increased during the 24 h time period (Fig. 2a, b and Additional file 6). In line with our results from the immunostainings, very little of the Aβ_42_-555 protofibrils co-localized with neurons (identified by their oval cell bodies, distinct axons and active migration) (Fig. 2a, c and Additional file 6).

The tg-ArcSwe mouse model is known to have elevated levels of soluble Aβ aggregates including protofibrils, and AD-like Aβ plaque pathology with an onset around six months of age [39–41]. Immunostainings of brain sections from 12 to 14-month-old tg-ArcSwe mice, using antibodies against GFAP and Aβ demonstrated that astrocytes, as expected, were tightly localized around Aβ plaques (Fig. 2d) and that Aβ co-localize with astrocytes in vivo (Additional file 7). Interestingly, we also found that some of the Aβ that had been ingested by reactive astrocytes co-localized with condensed nuclei of dead cells, confirming that our findings in the cell cultures reflect cellular processes of Aβ pathology in vivo (Fig. 2e).

### Aβ protofibrils are accumulated in astrocytes for a very long time

**Fig. 3:**
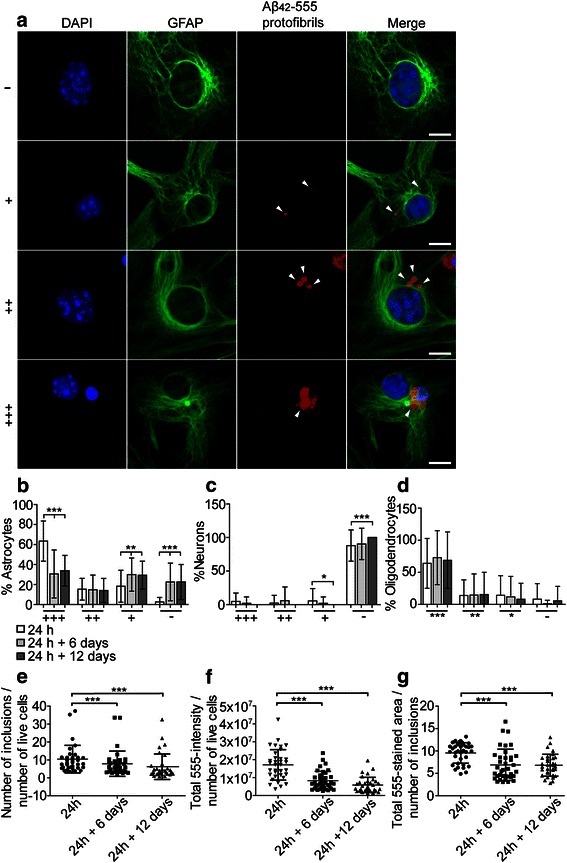
Aβ_42_ are stored in astrocytes for a very long time. To follow degradation of intracellular Aβ_42_-555 in astrocytes following Aβ_42_-555 protofibril removal, cells were divided into four categories; cells with no detectable levels of Aβ_42_-555 (–), cells with only small amounts of Aβ_42_-555 (+), cells containing medium sized Aβ_42_-555 deposits (++) and cells with large Aβ_42_-555 inclusions (+++) (**a**). Astrocytes degrade Aβ_42_-555 protofibrils very slowly and much Aβ_42_ remains in the cells 12 days after Aβ_42_-555 protofibril removal (**b**). Neurons contain almost no Aβ_42_ (**c**). The few oligodendrocytes in the culture also degrade the Aβ_42_-555 protofibrils slowly (**d**). Although the glial cells degrade the Aβ_42_-555 protofibrils slowly, the total number of 555-stained inclusions (24 h: 10.4 ± 7.7; 24 h + 6 days: 7.9 ± 7.1; 24 h + 12 days: 6.2 ± 7.1) in the culture decline significantly from 24 h to 24 h + 6 days (*P* = 0.013) and 24 h + 12 days (*P* < 0.001) (**e**). In line with these results, the total 555-intensity (24 h: 1.7 × 10^7^ ± 8.5 × 10^6^; 24 h + 6 days: 8.3 × 10^6^ ± 5.1 × 10^6^; 24 h + 12 days: 5.9 × 10^6^ ± 4.3 × 10^6^) (**f**) and total 555-positive area (24 h: 9.6 ± 2.3 μm^2^; 24 h + 6 days: 6.7 ± 3.5 μm^2^; 24 h + 12 days: 6.8 ± 2.5 μm^2^) (**g**) also decline significantly over time (*P* < 0.001 already from 24 h to 24 h + 6 days). Aβ inclusions are marked with white arrow heads. Scale bars: 10 μm. The experiments were performed in triplicates with independent cell cultures and 10 images/experiment were analyzed. Mann–Whitney *U*-test ***P < 0.001

To follow the degradation of intracellular Aβ_42_ protofibrils in astrocytes, neurons and oligodendrocytes, cell cultures were thoroughly washed after the 24 h Aβ_42_-555 protofibril exposure and cultured for additional 6 or 12 days prior to fixation and staining. The cells were divided into four categories; cells with no detectable levels of Aβ_42_-555 (−), cells with only small amounts of Aβ_42_-555 (+), cells containing medium sized Aβ_42_-555 inclusions or larger Aβ_42_-555 inclusions with low intensity (++) and cells with one or more large Aβ_42_-555 inclusion with high intensity (+++). The four categories in combination with GFAP staining are shown in Fig. 3a. Quantification of the percentage of astrocytes in each category demonstrates that most astrocytes contained large inclusions of Aβ_42_-555 (+++) directly after the 24 h exposure (63.5 ± 20.1 %). Although the percentage of (+++) astrocytes had decreased significantly after 6 days (30.7 ± 24.0 %, *P* < 0.001) and 12 days (33.8 ± 15.3 % *P* < 0.001), the degradation was extremely slow. The percentage of astrocytes containing medium sized Aβ_42_-555 inclusions (++) was lower at 24 h (15.4 ± 11.0 %), but did not decrease over time. Astrocytes containing small amounts of Aβ_42_-555 (+) and astrocytes with no detectable levels of Aβ_42_-555 (−) increased significantly from 24 h (18.3 ± 16 % respective 2.6 ± 4.6 %) to 24 h + 6 days (30.0 ± 16.6 %, *P* = 0.0034 respective 22.6 ± 18.8 %, *P* < 0.001). Taken together, these results demonstrate that astrocytes effectively ingest Aβ_42_ protofibrils, but that the ingested material is only partially degraded within 12 days (Fig. 3b). Neurons had low or no detectable Aβ_42_-555 inclusions at 24 h (Fig. 3c) whereas most of the few oligodendrocytes in the culture contained large (+++) or medium (++) Aβ_42_-555 inclusions that did not significantly change over time (Fig. 3d). As a complement, we next analyzed the total number of inclusions, total intensity and total area of Aβ_42_-555 deposits (normalized to the total number of cells) in the cell culture, using the Zen 2012 software. The number of 555-stained inclusions, normalized to the number of live cell nuclei, declined significantly from 24 h to 24 h + 6 days (*P* < 0.001), indicating that the inclusions were either degraded or fused (Fig. 3e). The total 555-intensity (Fig. 3f) and total 555-positive area (Fig. 3g) also declined significantly over time (*P* < 0.001 already from 24 h to 24 h + 6 days), confirming that the astrocytes degrade the Aβ_42_-555 protofibrils to some degree, although very slowly. In contrast to our results with Aβ_42_-555 protofibrils, cell cultures exposed to Aβ_40_-555 monomers, showed no accumulation of Aβ in astrocytes (Additional file 8).

### Engulfed Aβ protofibrils co-localize with the lysosomal marker LAMP-1

**Fig. 4:**
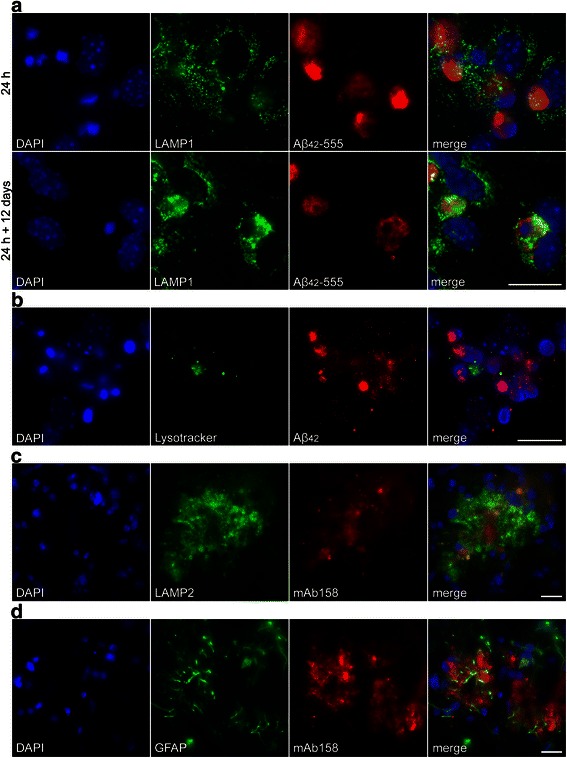
Aβ_42_ accumulates in immature lysosomes. The co-localization of Aβ_42_ and LAMP-1 positive lysosomes increases from 24 h to 24 h + 12 days (**a**). LysoTracker staining does not overlap with the Aβ_42_ protofibril inclusions, demonstrating that the Aβ_42_ containing lysosomes are immature (**b**). Aβ_42_ protofibrils co-localize with LAMP-2 (**c**) and GFAP (**d**) in sections of 14-month-old tg-ArcSwe mice. Scale bars: **a**–**d** = 20 μm

In order to investigate the intracellular localization of the Aβ deposits in astrocytes we next performed immunostainings with the lysosome specific antibody LAMP-1. Our results show that there was only little overlap between LAMP-1 and Aβ_42_ directly after the Aβ_42_ protofibril exposure (24 h), but the co-localization increased over time and 12 days after Aβ_42_ protofibril removal, most of the intracellular Aβ_42_ deposits co-localized with LAMP-1 positive vesicles (Fig. 4a). These results demonstrate that the engulfed Aβ_42_ protofibrils were transported to lysosomal compartments within the astrocytes. However, we have previously shown that engulfed dead cells in astrocytes can be present in LAMP-1 positive phagosomes/lysosomes without being degraded. The reason is insufficient acidification of astrocytic lysosomes resulting in inadequate digestion of the ingested material [26]. To clarify if the lysosomes in the Aβ_42_ protofibril accumulating astrocytes were acidic or not, we performed experiments using LysoTracker, which is a dye that labels acidic organelles. We found that although being LAMP-1 positive, the Aβ_42_ containing lysosomes did not stain with the LysoTracker, indicating that Aβ_42_ stored in the glial cells were situated in immature lysosomes (Fig. 4b). Interestingly, immunostainings of brain sections from 14-month-old tg-ArcSwe mice using specific antibodies against LAMP-2 and mAb158 (Fig. 4c) or GFAP and mAb158 (Fig. 4d) demonstrated that Aβ co-localized with both LAMP-2 and GFAP in vivo. It was the outermost layer of the Aβ plaque, rather than the plaque core that was LAMP-2 positive, suggesting that the most superficial Aβ could actually be situated in lysosomal compartments inside the glial cells that tightly surround the plaque (as GFAP only stains 15–20 % of the astrocytic cell bodies [36]).

### Intracellular stored Aβ is truncated in the N-terminus

**Fig. 5:**
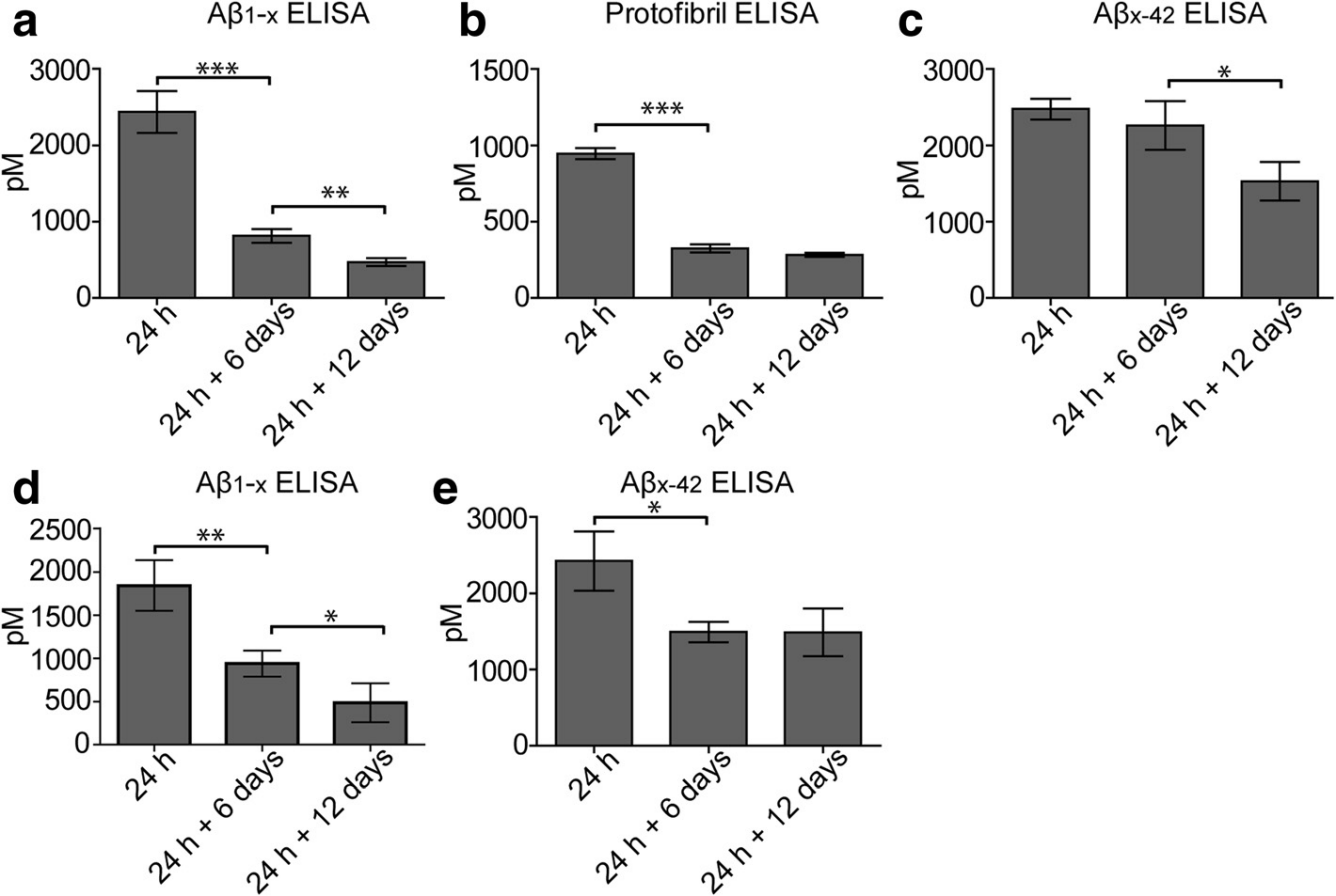
Intracellular stored Aβ_42_ is truncated in the N-terminus. Aβ ELISAs of cell lysates from 24 h and from 6 to 12 days following Aβ_42_ protofibril removal (24 h + 6 days and 24 h + 12 days, respectively) demonstrate that a high proportion of the accumulated Aβ is N-terminally truncated. The concentrations of Aβ_1-x_ (**a**) and Aβ protofibrils (**b**) decrease continuously from 24 h (2434.0 ± 272.0 and 946.9 ± 36.5, respectively) to 24 h + 6 days (862.8 ± 92.0 and 325.8 ± 25.9, respectively) and 24 h + 12 days (468.2 ± 52.2 and 282.9 ± 13.1, respectively), while the concentrations of Aβ_x-42_ remain higher over time (24 h: 2475.0 ± 134.0, 24 h + 6 days: 226.01 ± 318.3 and 24 h + 12 days: 1531.0 ± 253.1) (**c**). In the pellets, remaining after the cell lysis procedure, the Aβ_1-x_ concentrations decline over time (24 h: 1840.0 ± 289.5, 24 h + 6 days: 938.3 ± 159.7, 24 h + 12 days: 483.3 ± 225.3) (**d**), while the Aβ_x-42_ concentrations are higher at 24 h (2422.0 ± 387.0) and more stable over time, 24 h + 6 days (1493.0 ± 132.7) and 24 h + 12 days (1488.0 ± 312.6) (**e**). All concentrations are expressed in picomolar (pM) units. Mean values are from duplicates of three independent experiments

We next sought to investigate if the Aβ_42_ stored in the immature lysosomes was partly degraded. For this purpose we analyzed cell culture lysates using three different sets of sandwich ELISAs; the N-terminus dependent Aβ_1-x_ ELISA, the protofibril specific mAb158 ELISA and the N-terminus independent Aβ_x-42_ ELISA. In the Aβ_1-x_ ELISA, one of the antibodies is specific for the N-terminal Aβ neo-epitope (82E1, raised against Aβ_1-16_) and the other antibody binds to the central region of Aβ (4G8, raised against Aβ17-24), measuring Aβ_42_ with an intact N-terminal. In the Aβ_x-42_ ELISA, one of the antibodies is specific for the C-terminal Aβ_42_ neo-epitope [42] and the other antibody binds to the central region of Aβ (4G8), measuring both intact and N-terminally truncated forms of Aβ. Both the Aβ_1-x_ ELISA and the mAb158 ELISA, showed a 3-fold decrease in Aβ concentrations from 24 h to 24 h + 6 days (*P* < 0.001 for both) (Fig. 5a, b). The Aβ levels measured by the Aβ_1-x_ ELISA decreased further and were halved at 24 h + 12 days compared to 24 h + 6 days (*P* = 0.0058) (Fig. 5a). In contrast, when analyzing the cell lysates using the Aβ_x-42_ ELISA, the concentrations did not decrease during the first 6 days and there was only a 1.5-fold decline from 24 h + 6 days to 24 h + 12 days (*P* = 0.036). Hence, the concentrations remained higher using the Aβ_x-42_ ELISA compared to the Aβ_1-x_ and mAb158 ELISA, indicating that a high proportion of the stored Aβ is N-terminally truncated (Fig. 5a–c). Since our immunostainings demonstrated that the engulfed Aβ often co-localized with condensed cell nuclei of dead cells that had also been ingested by the astrocytes (Fig. 1a), we performed additional analysis of the pellet (consisting of nuclei and cell debris) that remained after the cell lysis procedure. The pellets were treated with formic acid and sonication to extract Aβ prior to analysis with the Aβ_1-x_ ELISA and the Aβ_x-42_ ELISA. Interestingly, we found that the pellet fraction from 24 h, but also from 24 h + 6 days and 24 h + 12 days showed higher Aβ concentrations in the Aβ_x-42_ ELISA compared to the Aβ_1-x_ ELISA, indicating existence of N-terminally truncated Aβ_42_ (Fig. 5d, e). Taken together, the results show that full length Aβ_1-42_ and Aβ_42_ protofibrils (Fig. 5a, b, d) decrease more over time than N-terminally truncated forms of Aβ (Fig. 5c, e), indicating that the intracellular deposits of Aβ in astrocytes are processed by the astrocytes in a way that may change their properties and toxicity.

### Aβ accumulation induces formation of giant astrocytic endosomes

**Fig. 6:**
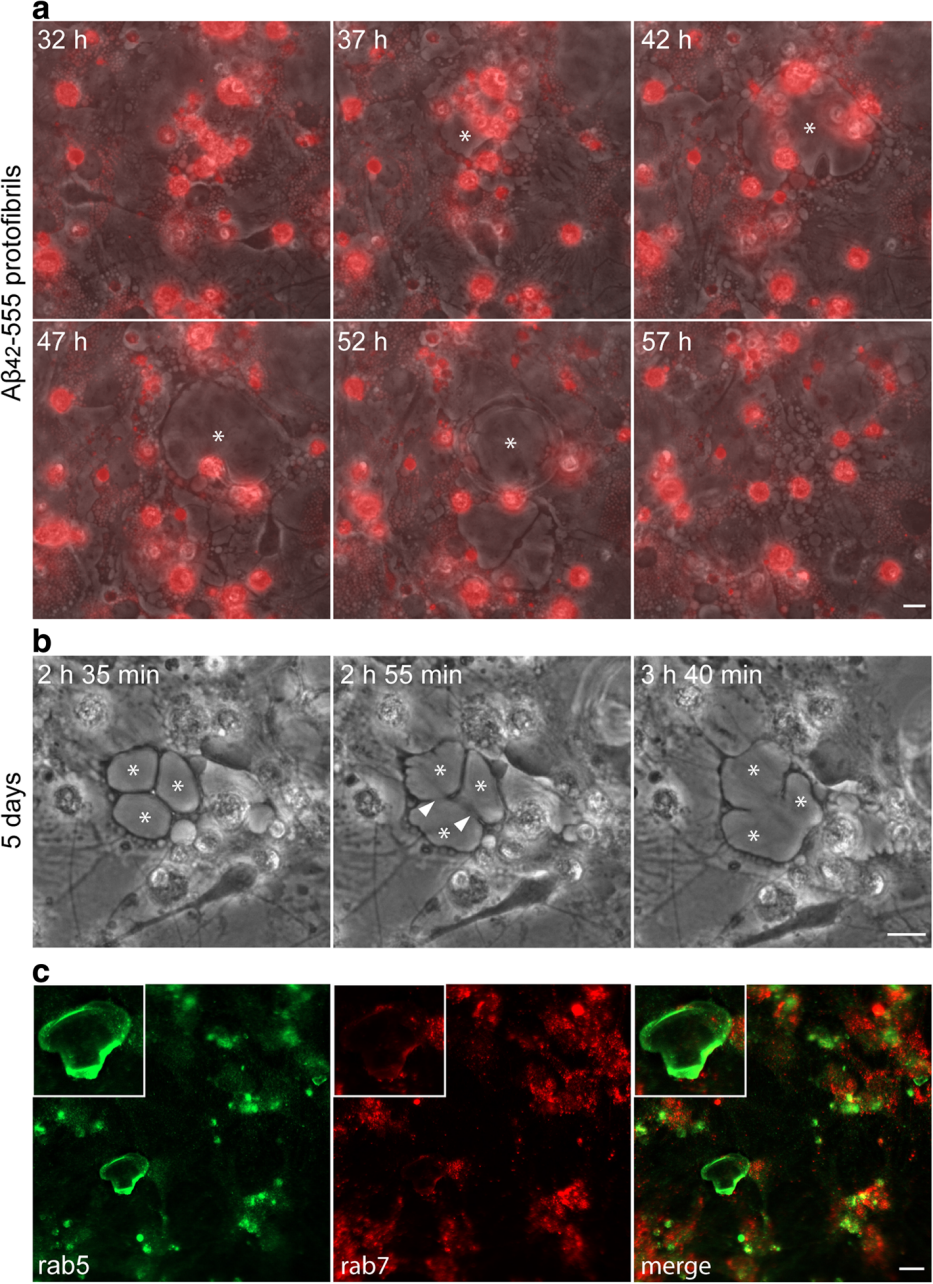
Aβ_42_ protofibril treatment results in formation of enlarged endosomes. Time-lapse experiments demonstrate that Aβ_42_ protofibril treatment induces the formation of enlarged, dynamic vacuoles (*) in the astrocytes (**a**). The enlarged vacuoles fuse (white arrow heads) with adjacent vacuoles; forming giant vacuoles with a diameter of 30–50 μm (**b**). Immunocytochemistry show that the vacuoles express the early endosome marker Rab5 and to a lesser extent the late endosome marker Rab7 (**c**). Inset in left corner of image **c** shows higher magnification of relevant structure. Scale bars: **a**–**b** = 10 μm, **c** = 20 μm

The cell cultures were followed for up to 5 days after the Aβ_42_ protofibril exposure, using time-lapse recordings. Interestingly, we found that the accumulation of Aβ_42_ protofibrils induced formation of enlarged, dynamic vacuoles in the astrocytes (Fig. 6a, and Additional file 9). The vacuoles increased rapidly in size, fused with adjacent vacuoles and resulted in giant vacuoles with a diameter of ~50 μm (Fig. 6b, and Additional file 10). The giant vacuoles eventually shrank or collapsed, but concurrently new vacuoles appeared in the same cell (Fig. 6a, and Additional file 9). The enlarged vacuoles were never found in control cultures, indicating that they are a direct result of the high Aβ_42_ load in the astrocytes. Immunostainings of fixed, Aβ_42_ protofibril exposed cell cultures, using specific antibodies to the early endosomal marker, Rab5, and the late endosomal marker, Rab7, demonstrated that the giant vacuoles are proposed to be derived from early endosomes (Fig. 6c). Moreover, double stainings with specific antibodies to Aβ/Rab5 and Aβ/Rab7 (Additional file 11), showed a clear co-localization of Aβ inclusions and Rab5. Some co-localization was also noted for Aβ and Rab7. These data emphasize that accumulation of Aβ_42_ protofibrils induces severe lysosome failure in the phagocytic astrocytes. The collapsing vacuoles did not cause astrocytic cell death during the time-lapse experiment, but may induce the release of Aβ containing microvesicles from the astrocytes.

### Engulfing astrocytes induce secondary Aβ toxicity

**Fig. 7:**
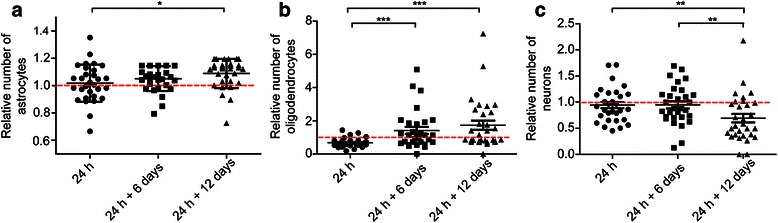
Aβ_42_ protofibril treatment induces secondary neuronal cell death. The number of astrocytes in Aβ_42_ protofibril exposed cultures is significantly increased from 24 h (1.0 ± 0.1) to 24 h + 12 days (1.1 ± 0.1, *P* = 0.011) (**a**). Similar to astrocytes, the number of oligodendrocytes increases from 24 h (0.7 ± 0.3) to 24 h + 12 days (1.7 ± 1.5, *P* < 0.001) in Aβ_42_ protofibril treated cultures (**b**). Aβ_42_ protofibril treatment has no direct effect on neurons, but the neuronal cell number significantly decreases (from both 24 h, 0.9 ± 0.4, *P* = 0.0061 and 24 h + 6 days, 1.0 ± 0.4, *P* = 0.0083) in the treated cultures 12 days (0.7 ± 0.5) after Aβ removal (**c**). The relative number of viable cells in Aβ_42_ protofibril exposed cultures is normalized to viable cells in unexposed cultures. The experiments were performed in triplicates with independent cell cultures and 10 images/experiment were analyzed. Mann–Whitney *U*-test ***P < 0.001

To further investigate how the Aβ_42_ protofibril exposure affects viability of the three different cell types in the culture, we quantified the number of living astrocytes, neurons and oligodendrocytes directly after the 24 h Aβ_42_ protofibril exposure and 6 and 12 days after Aβ_42_ protofibril removal. The total number of cells was compared to the cell number in parallel control cultures that did not receive any Aβ_42_ protofibrils. A modest, but significant, increase in the cell number following Aβ_42_ protofibril exposure was noted at 24 h + 12 days, for both astrocytes and oligodendrocytes (*P* = 0.011 and *P* < 0.001, respectively) (Fig. 7a and b), indicating that the Aβ_42_ protofibril clearing process induces some proliferation of the glial cells. In contrast, the number of neurons was decreasing over time in the Aβ_42_ protofibril exposed cultures (Fig. 7c). The fact that Aβ_42_ protofibril exposure did not induce neuronal death directly, but the decrease in neuronal number appeared 12 days after Aβ_42_ protofibril removal (*P* = 0.0061), indicates that the cell death is due to a secondary mechanism.

### Neuronal cell death is induced by secreted microvesicles

**Fig. 8:**
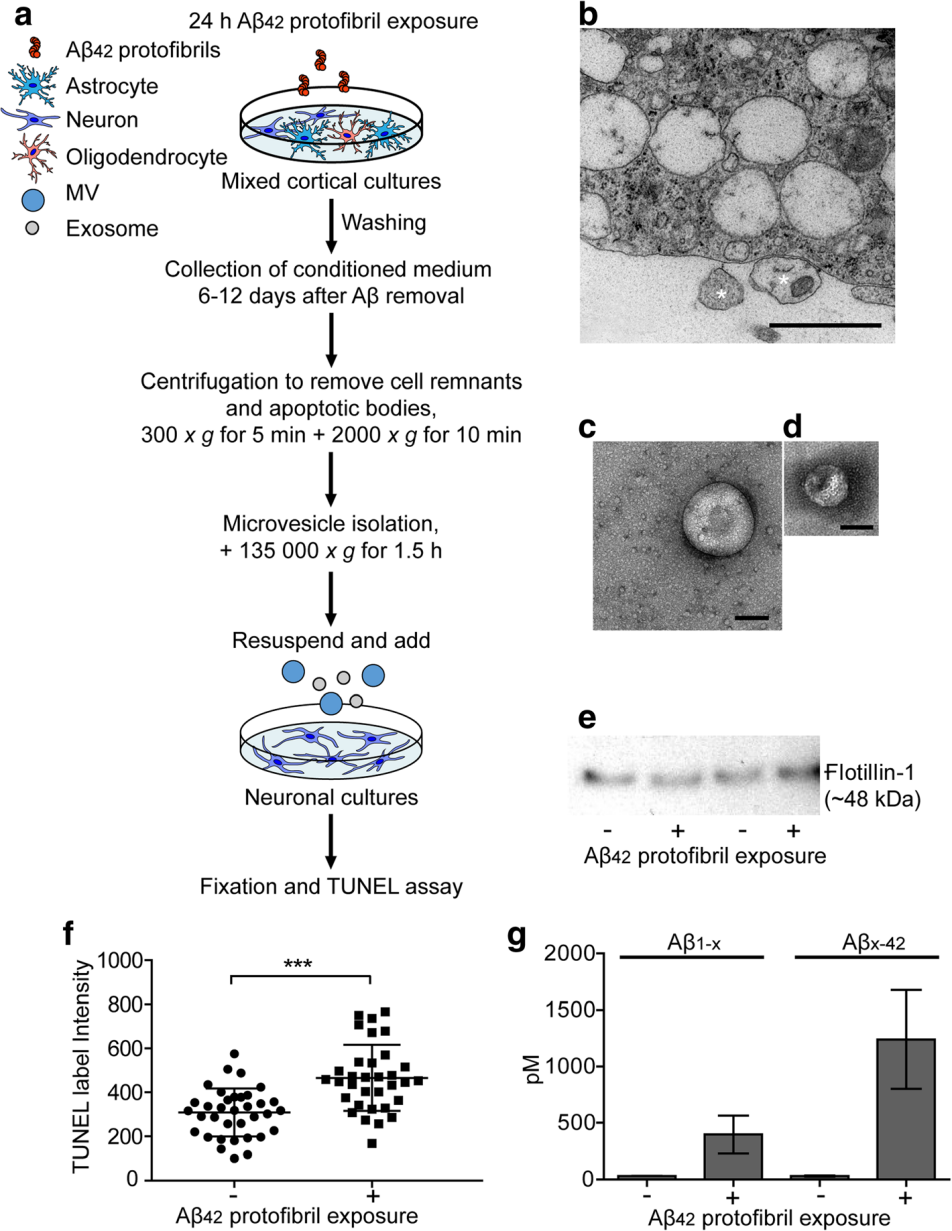
Neuronal cell death is induced by secreted microvesicles. Illustration of the experimental setup (**a**). Electron microscopy images of Aβ_42_ protofibril exposed co-cultures demonstrate microvesicle (*) secretion from a single astrocyte (**b**) and a larger (**c**) and smaller (**d**) microvesicle present in the cell culture medium. Western blot analysis show that the isolated microvesicles express Flotillin-1, but that the levels are unchanged in Aβ_42_ protofibril treated cultures, compared to controls (**e**). TUNEL assays demonstrate a significant increase (*P* < 0.001) in apoptotic neurons following treatment with microvesicles from Aβ_42_ protofibril exposed co-cultures (465.4 ± 150.4), compared to microvesicles from untreated co-cultures (308.3 ± 109.2) (**f**). The experiments were performed in triplicates with independent cell cultures and 10 images/experiment were analyzed. Microvesicles isolated from medium collected 6 and 12 days after Aβ_42_ protofibril exposure contain Aβ as revealed in Aβ_1-x_ (396.9 ± 167.2 pM) and Aβ_x-42_ (1239.0 ± 438.7 pM) ELISA. The Aβ_1-x_ concentration is lower than Aβ_x-42_, indicating that there is a truncation of the Aβ_42_ N-terminus (**g**). Duplicate samples from 3 independent experiments were analyzed. Mann–Whitney *U*-test ***P < 0.001. Scale bars: **b** = 1 μm, **c**–**d** = 100 nm

To identify possible mechanisms for the secondary toxicity, we investigated if microvesicles secreted by Aβ_42_ protofibril treated co-cultures induce apoptosis of cortical neurons. For these experiments we exposed co-cultures with Aβ_42_ protofibrils for 24 h, washed the cultures thoroughly and cultured the cells in Aβ_42_ protofibril-free medium for additional 12 days. Parallel control cultures received medium only. Microvesicles were isolated using ultracentrifugation of conditioned medium harvested day 6 and 12 following Aβ_42_ protofibril removal. The microvesicle pellets were reconstituted in neurobasal medium and added to embryonic cortical mouse neurons that had been cultured for 12 days (12 days in vitro). After 48 h, the neurons were fixed and the number of apoptotic, TUNEL labeled neurons was compared (Fig. 8a). Studies of astrocytes using electron microscopy, demonstrated a release of microvesicles of different sizes (Fig. 8b). Moreover, electron microscopy analysis of the microvesicle preparations, identified microvesicles (>100 nm in diameter, Fig. 8c–d). Microvesicles, isolated from both untreated and Aβ_42_ protofibril exposed cell cultures, expressed the microvesicle marker Flotillin-1. However, no differences in the total protein concentration of Flotillin-1 was detected between untreated and Aβ_42_ protofibril exposed cell cultures (Fig. 8e). Interestingly, there was a significant increase in apoptotic neurons in cultures treated with microvesicles from Aβ_42_ protofibril exposed co-cultures compared to controls (Fig. 8f), demonstrating that Aβ_42_ protofibril accumulation induces secretion of microvesicles with toxic content. Parallel neuronal cultures, exposed to intact Aβ_42_ protofibrils, directly added to the medium, showed no difference in the percentage of TUNEL positive cell nuclei or the total number of cell nuclei, compared to untreated cultures (Additional file 12). Since the amount of microvesicles was unchanged (based on the Flotillin-1 expression), the apoptotic effect on neurons was due to the content of the microvesicles. Aβ_1-x_ and Aβ_x-42_ ELISA analysis of microvesicles isolated from the cell culture medium demonstrate that the microvesicles contained primarily an N-terminally truncated form of Aβ_42_ (Fig. 8g).

## Discussion

Many studies indicate that the widespread neuronal dysfunction in the AD brain is caused by soluble Aβ aggregates, such as protofibrils, rather than the insoluble fibrils [8–13]. The mechanisms by which Aβ_42_ protofibrils induce neuronal toxicity is however unclear. It has been suggested that Aβ oligomers promote neuronal cell death, inhibit long-term potentiation and impair synaptic function and plasticity [8, 9, 11, 12, 43–46]. In the present study we show, by using a co-culture system of neurons and glia, that Aβ_42_ protofibrils are not directly, but indirectly neurotoxic. Extensive engulfment of Aβ_42_ protofibrils by the astrocytes in the co-culture results in long-term intracellular deposits of Aβ, severe lysosomal dysfunction and formation of N-terminally truncated Aβ that is spread in the co-culture by microvesicle secretion.

Accumulation of Aβ in the AD brain is due to an imbalance between the Aβ production and Aβ clearance. Several studies indicate that monomeric Aβ_40_ and Aβ_42_ are degraded by cultured astrocytes following ingestion [47–49]. This conclusion was based on observations that the engulfed Aβ was directly transported to LAMP-positive lysosomes following ingestion [47, 49, 50]. However, neither of the studies confirmed if Aβ degradation actually took place in the lysosomes. We hypothesized that aggregated species of Aβ, such as protofibrils, may be more difficult for the cells to handle than the monomeric form of the protein. Our results show that the degradation of Aβ_42_ protofibrils in astrocytes is indeed extremely slow, although the ingested Aβ is situated in LAMP-1 phagosomes/lysosomes. Instead, the Aβ_42_ protofibrils accumulate in the astrocytes, leading to large, intracellular inclusions. In contrast to Aβ_42_ protofibrils, Aβ_40_ monomers were not accumulated in the astrocytes, indicating that they are not overall slow degraders. We have previously compared phagocytosis of dead cells by astrocytes and macrophages (which are indistinguishable from activated microglia in the pathological brain) and found that astrocytes in contrast to the professional phagocytes store, rather than degrade ingested dead cells [26, 30]. By using the pHrodo-labeling technique we demonstrated that material ingested by macrophages fuses with mature/acidic lysosomes within 5 h, whereas material engulfed by astrocytes still had not fused with acidic lysosomes after 3 days. Thus, astrocytes are ineffective when it comes to degrading large/toxic material such as cell corpses and protein aggregates. Some reports indicate that astrocytes may be more efficient than microglia in taking up Aβ, particularly during the early stages of AD and astrocytes has been shown to gradually accumulate Aβ throughout cortex in AD patients [28, 29, 51–53]. However, the difference in degradation capacity of the two cell types has not been taken in account in these studies.

Our data show that glial cells, but not neurons accumulate Aβ_42_ protofibrils. Since the glial cells in our differentiated co-culture system do not die following Aβ exposure, it is unlikely that the noted co-localization of Aβ and dead nuclei is due to phagocytosis of dead Aβ containing cells. Dead cells are however constantly ingested by the astrocytes during the differentiation process, when apoptosis is naturally occurring. These dead cells are then stored in the astrocytes for weeks. Hence, the co-localization is probably due to transportation of the ingested material to the same “garbage dumps” within the astrocytes. Moreover, the ingestion of Aβ is very rapid. Our time-lapse experiments demonstrated that intracellular Aβ-555 deposits are visible already after 30 min (Fig. 2a). Over time the 555-signal of these deposits becomes more and more intense, indicating that the Aβ aggregates are constantly engulfed by the astrocytes and then transported to specific sites within the astrocytes.

In an attempt to identify reasons for the slow digestion of engulfed dead cells in primary astrocytes, we previously found that actin rings surround the phagosomes for long periods of time, which physically inhibit the phago-lysosome fusion [30]. Furthermore, astrocytes express high levels of Rab27a, a protein known to reduce the acidity of lysosomes by Nox2 recruitment, in order to preserve antigens for presentation. We demonstrated that Nox2 co-localizes with the ingested material, indicating that it may influence antigen processing also in astrocytes, as they express MHC class II [30]. By inducing long-time acidification of astrocytic lysosomes using acidic nanoparticles, we could increase the digestion of astrocyte-ingested dead cells. The degradation was, however, normalized over time, indicating that inhibitory pathways are up-regulated in response to the enhanced acidification [30]. By staining the cell cultures with LysoTracker dye in the present study, we also found the lysosomes to be of low acidity, which probably contributes to the incomplete digestion of Aβ. Moreover, the deposition of aggregated Aβ_42_ in astrocytes results in severe lysosomal dysfunction, including formation of enormous endosomes, indicating that the accumulation of Aβ disturbs the endosome/lysosome machinery. Such enlarged endosomes were never observed in astrocytes storing cell corpses, or cultures exposed to Aβ_40_ monomers, but only in Aβ_42_ protofibril accumulating astrocytes. It is likely that this toxic effect of Aβ_42_ protofibrils affects the astrocytes so that their slow degradation is further reduced.

The fact that the Aβ_42_ deposits in astrocytes co-localize with the lysosomal marker LAMP-1 indicates that the lysosome function is crucial for Aβ degradation. It is however not excluded that other degradation mechanisms, such as the proteasome machinery could be affected by the presence of toxic Aβ aggregates in the cell. Sections of tg-ArcSwe mice revealed that the Aβ plaques were surrounded by LAMP-2 immunoreactive astrocytes. These findings agree with previous reports of increased LAMP-1 in cerebral cortex of AD patients as the disease progresses [54] and in brains of APPSL-Tg mice during aging [55]. Interestingly, it has been demonstrated that activation of transcription factor EB (TFEB), a regulator of lysosome biogenesis, enhanced uptake and degradation of Aβ in astrocytes, attenuating plaque pathology in APP/PS1 transgenic mice [49]. By studying the lysosomal storage disorder, multiple sulfatase deficiency (MSD), it has been suggested that normal autophagic, endocytic, and lysosomal vesicle trafficking is essential for preventing neurodegeneration [56]. One of the earliest pathological signs observed in patients with AD is the accumulation of numerous enlarged autophagic and endosomal vesicles, due to defective autophagy-lysosomal degradation in neurons [33, 57, 58]. The defect may result from impaired vesicle fusion among autophagosomes, endosomes and lysosomes [34, 57, 59, 60] or failure of lysosomal acidification [61]. Aβ_42_ has been shown to induce autophagy and lysosomal degradation dysfunctions which may lead to indigestible Aβ storage inside the vesicles [62, 63].

The accumulated Aβ in the astrocytes was partly modified to N-terminally truncated Aβ. The absence of the full Aβ N-terminus may thereby, to some extent, underestimate the intracellular Aβ concentration when analyzing cell lysate with the N-terminus dependent Aβ_1-x_ and Aβ protofibril ELISA. N-terminally truncated Aβ, often bearing amino-terminal Glu3 which eventually modifies to pyroglutamate (AβN3pE-x), is found both in extracellular, intracellular and vascular deposits in AD and Down's syndrome brain tissue [64]. Astrocytes have been implied to take up N-terminally truncated Aβ from fleecy amyloid in AD brains and diffuse plaques from non-demented individuals [65, 66]. As we exposed astrocytes with full length Aβ_42_ in the protofibrillar form, we propose that the truncation of Aβ in fact might occur by the endosomal/lysosomal pathway in the astrocytes. Since the N-terminals are sticking out from the Aβ protofibrils, they are easily accessible, compared to the C-terminals that are hidden in the core of the aggregate. We suggest that since the astrocytes degradation system is ineffective, degradation is not completed and partly degraded (N-terminally truncated) Aβ is left. N-terminally truncated Aβ has been found to be more resistant to degradation, more prone to aggregate and more toxic than full - length Aβ [67]. Our hypothesis is that astrocytes try to be “helpful”, but are overwhelmed by the difficulties they face. Hence, they cannot fulfill their task to degrade the Aβ protofibrils, and instead of being “helpful” they promote spreading of the Aβ pathology, by secreting Aβ aggregates that had been only partly degraded. In the AD brain, reactive astrocytes are attracted to the Aβ plaques and are highly exposed to various forms of Aβ aggregates. Engulfment of Aβ by astrocytes may initially be a protective clearance mechanism, but based on our data, overburden of the astrocytes is clearly detrimental.

Astrocytes are highly responsible for maintaining brain homeostasis and although our results show that the high Aβ load does not induce apoptosis in astrocytes, their normal functions are probably severely affected. Misconduct in the performance of astrocytes normal responsibilities would affect processes such as metabolic support of neurons, modification of synapse signaling, recycling of neurotransmittors, regulation of blood flow and function of the blood brain barrier [2, 3].

The way in which Aβ pathology spreads in the AD brain has been debated for many years. Experimental evidence suggests that amyloid proteins, such as Aβ, indeed can seed and transmit pathology in the brain. For example, it has been shown that injection of cortical extracts from post-mortem AD brains into the brains of transgenic APP mice, aggravates plaque pathology within five months [68] and longer incubation also induces tau pathology [69]. The cellular mechanism behind the spreading of AD pathology is still unclear, but both cell-to-cell transmission [70] and secretion into the extracellular space [71] have been suggested as possible pathways. Ineffective degradation of Aβ may lead to spreading of AD pathology, due to secretion of Aβ containing microvesicles [35, 72]. Interestingly, Aβ peptides have been found to be released from AβPP transfected neuroblastoma cells via exosomes. Moreover, the exosome specific proteins Alix and Flotillin-1, have been found around plaques in human AD brains and within senile plaques of Tg2756 mice, respectively [72, 73]. We have previously shown that astrocytes express high levels of proteins known to promote vesicle secretion [26, 30, 74, 75]. In contrast to neurons, which mainly secrete Aβ beginning at Asp1, the majority of Aβ secreted from glial cells has proven to be N-terminally truncated [76].

## Conclusion

In conclusion, we demonstrate that astrocytes engulf large amounts of protofibrillar Aβ_42_ that are only partly digested and stored in the cells for very long times. The intracellular Aβ accumulation results in severe astrocytic endosome/lysosome defects and microvesicle-induced neurotoxicity. In familial AD, with mutations in the AβPP, *Presenilin 1* or *Presenilin 2* genes, increased Aβ production or increased Aβ_42_/Aβ_40_ ratio lead to AD. However, in sporadic AD it is likely that defective Aβ clearance is the culprit. Thus, accumulation of Aβ in astrocytes could play a vital role in the sporadic form of the disease and a better understanding of astrocytes role in AD initiation and progression is highly desirable.

## Methods

### Synthetic Aβ_42_ protofibrils

**Fig. 9:**
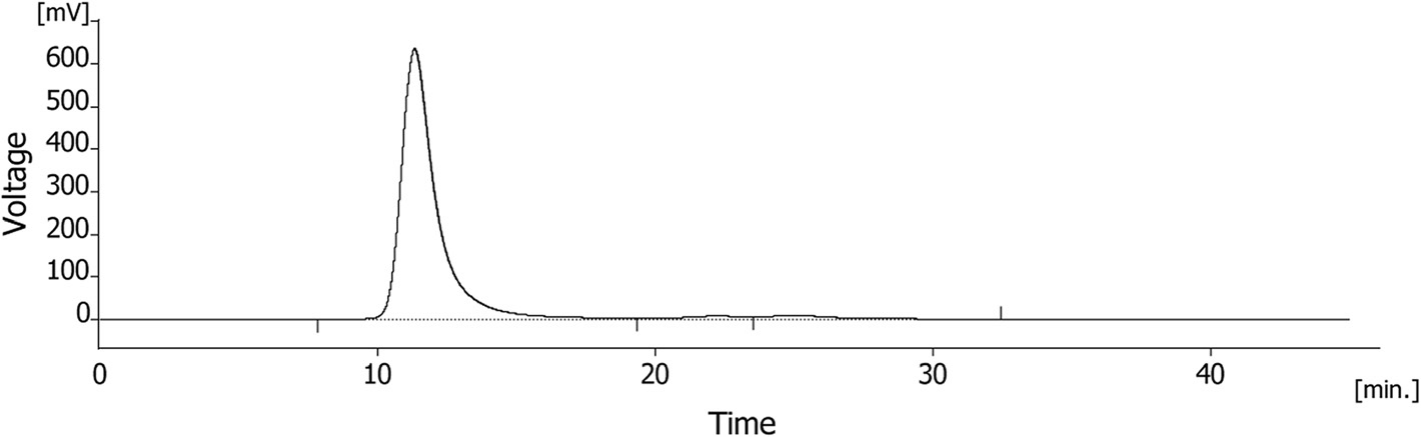
Aβ_42_ protofibril characterization. A chromatogram following Aβ_42_ protofibril analysis by SEC using a Superdex 75 column. The chromatograms show mV for the absorbance at 214 nm on the *y-axis* and the retention time in minutes on the *x-axis*

Fluorescent HiLyte™ Fluor 555-labeled Aβ_42_ (Aβ_42_-555) peptides (Anaspec Inc) were diluted in 10 *x* phosphate buffered saline (PBS) to a concentration of 36 μM followed by incubation for 4 h at 37 °C. Synthetic Aβ_42_ peptides (American Peptide Company Inc.) were prepared as previously described [13, 77–79]. Aβ_42_ dissolved in 10 mM NaOH was mixed with 10 *x* PBS to 443 μM (2 mg/ml) and incubated 30 min at 37 °C. Both Aβ_42_-555 protofibrils and unlabeled Aβ_42_ protofibrils were centrifuged for 5 min at 17 900 *x g* to remove any insoluble aggregates. Using the protofibril specific ELISA, mAb158 [41], we concluded that we had the best yield of Aβ_42_-555 protofibrils after 4 h incubation in 37 °C. Aβ_42_-555 protofibrils were readily detected by mAb158 ELISA and there was no significant difference between 555-labeled and unlabeled Aβ_42_ protofibrils (Additional file 13). To estimate the purity (>95 %) and size of the Aβ_42_ protofibrils, 50 μl of 250 μg/ml Aβ_42_ protofibrils were analyzed by size-exclusion chromatography (SEC) using a Superdex 75 column. The Aβ_42_ protofibrils (>95 % purity) eluted in the void volume and was estimated to be >75 kDa based on the cutoff size of the Superdex column (Fig. 9).

### Animals

All experiments involving animals were performed at Uppsala University, Sweden. The experiments were approved by the Uppsala County Animal Ethics Board (ethical permit number: C75/13, valid 2013-06-28 to 2018-06-28), following the rules and regulations of the Swedish Animal Welfare Agency, in compliance with the European Communities Council Directive of 22 September 2010 (2010/63/EU). C57/BL6 mice were used for cell culture studies and tg-ArcSwe mice, harboring the human *Arctic (E693G)* and *Swedish (KM670/671NL)AβPP* mutations (Lord 2007) for in vivo experiments. The animals were housed at the National Veterinary Institute, Uppsala or the animal facility at Uppsala University Hospital, Uppsala in a 12-12 dark-light cycle. The mice were kept in an enriched environment and given water and food *ad libitum*.

### Neural cell cultures

Cerebral cortices from C57/BL6 mice of embryonal day 14 (E14) were dissected in Hank’s buffered salt solution supplemented with 50 U/ml Penicillin, 50 mg/ml Streptomycin and 8 mM Hepes buffer (HBSS, all from Invitrogen). The cortices were dissociated in fresh HBSS, centrifuged at 150 *x* g and resuspended in cell culture medium.

#### Co-cultures of neurons and glia

According to Loov et al. [30], the cells were expanded in DMEM/F12-GlutaMAX supplemented with 1 *x* B27, 50 U/ml Penicillin, 50 mg/ml Streptomycin and 8 mM Hepes buffer, 10 ng/ml bFGF (all from Invitrogen) and 20 mg/ml EGF (VWR). Neurospheres were passaged every second or third day by dissociation in HBSS and resuspended in medium with bFGF and EGF. Prior to experiments, the cells were plated as a monolayer, at a concentration of 1.5 × 10^5^ cells/ml, on cover slips (In Vitro Diagnostics) or cell culture dishes (Falcon), coated with Poly-L-Ornithine (Sigma-Aldrich) and Laminin (Invitrogen). After 24 h, the medium was replaced with mitogen-free medium to initiate neural stem cell differentiation to a mixed population of neurons, astrocytes and oligodendrocytes, but not microglia. This is a well characterized cell culture system, based on the lineage restricted differentiation of embryonic, cortical stem cells [38, 80, 81]. To drive the differentiation towards generation of exclusively astrocytes, 10 ng/ml cilliary neurotrophic factor (CNTF) was added to the mitogen-free medium throughout the differentiation process [37, 38]. During the seven days differentiation period, the cell culture medium was changed every second or third day. Only neurospheres from passage 2–4 were used for experiments.

#### Neuronal cultures

Following dissection and dissociation the cells were seeded on Poly-L-Ornithine and Laminin coated cover slips at a concentration of 8 × 10^4^ cells/ml. The neurons were cultured in neurobasal medium supplemented with 1 *x* B27, 50 U/ml Penicillin, 50 mg/ml Streptomycin and 20 mM L-glutamine (Invitrogen) for 12 days (12 DIV) prior to experiment. The first day after seeding, the cell culture medium was fully replaced followed by changing half of the medium every second or third day.

### Aβ stimulation

Cell cultures differentiated into neurons and glia or astrocytes and pure neuronal cultures were treated with 0.1 μM Aβ_42_ protofibrils (either 555-labeled or unlabeled) for 24 h. Controls received fresh cell culture medium without Aβ_42_ protofibrils. After Aβ treatment, the cells were washed in cell culture media *x* 3 and the cover slips were transferred to new culture dishes. The cells were fixed, lysed (24 h) or cultured for additional 6 (24 h + 6 days) or 12 days (24 h + 12 days) in Aβ free - cell culture medium prior to fixation or cell lysis.

### Microvesicles

Co-cultures of neurons and glia were treated with 0.1 μM Aβ_42_ protofibrils for 24 h or left untreated. The cultures were thoroughly washed *x* 3 and were continuously cultured in medium only. From day 6 to day 12 following Aβ_42_ protofibril treatment, the medium was harvested and centrifuged for 5 min at 300 *x g* to remove any cell remnants. For microvesicle preparations (containing exosomes and larger vesicles), the supernatants were centrifuged for 10 min at 2000 *x g* to remove apoptotic bodies followed by ultracentrifugation for 1.5 h at 4 °C at 135 000 *x g*. The ultracentrifugation was performed in Ultra-Clear centrifuge tubes (Beckman Coulter) using a Beckman LE-80 ultracentrifuge. For the neurotoxicity assays, the pellets containing microvesicles were reconstituted in neurobasal medium supplemented with 1 *x* B27, Penicillin and Streptomycin and L-glutamine (Invitrogen) and added to cortical neurons. After 48 h, the neuronal cultures were fixed and the number of apoptotic cells quantified. Parallel control cultures were exposed to 0.1 μM Aβ_42_ protofibrils or left untreated for 48 h prior to fixation. For analysis of the Aβ content, the isolated microvesicles were lysed in ice cold lysis buffer (20 mM Tris pH 7.5, 0.5 % Triton X-100, 0.5 % deoxycholic acid, 150 mM NaCl, 10 mM EDTA, 30 mM NaPyroP and protease inhibitor (Roche)) and stored in −70 °C until time of analysis by ELISA.

### Immunostaining of cell cultures

Cover slips were fixed for 15 min in RT with 4 % paraformaldehyde and permeabilized and blocked with 0.1 % Triton X-100 (both from Sigma-Aldrich) and 5 % normal goat serum (NGS, Bionordica) in PBS for 30 min in RT. Primary antibodies were incubated in 0.1 % Triton X-100 with 0.5 % NGS for 1–4 h in RT or O/N in 4 °C. Thereafter, cover slips were washed thoroughly in PBS *x* 3 between each step. Incubation with secondary antibodies was performed in 0.1 % Triton X-100 and 0.5 % NGS for 45 min in 37 °C or 1 h in RT. The following primary antibodies were used in the study: rabbit anti-Glial Fibrillary Acidic Protein (GFAP, 1:400, DakoCytomation), mouse anti-GFAP (1:400, Sigma-Aldrich), mouse anti-2',3'-Cyclic-nucleotide 3'-phosphodiesterase (CNPase, 1:500, Sigma-Aldrich), mouse anti-βIII tubulin (1:200, Covance), rabbit anti-Lysosome-associated membrane protein-1 (LAMP-1, 1:200, Abcam), rabbit anti-Rab5 (1:1000, Abcam), mouse anti-Rab7 (1:1000, Abcam), polyclonal rabbit anti-Aβ_42_ (1:200, Invitrogen), monoclonal mouse anti-Aβ antibody, 6E10 (10 μg/ml, epitope: 3-8, Signet), the monoclonal mouse anti-Aβ antibody, mAb1C3 (10 μg/ml, epitope: 3-8) [41] and monoclonal mouse anti-Aβ protofibril selective antibody, mAb158 (10 μg/ml) [41]. Secondary antibodies used were: AlexaFluor 488, 555 and 647, all against mouse or rabbit (1:200, Molecular probes). To study acidic lysosomes, 0.5 μM LysoTracker red DND-99 (ThermoFisher) was added to the cell culture medium 2 h prior to fixation. Neuronal apoptosis was measured using terminal (TdT)-mediated dUTP-biotin reaction mixture (TUNEL, Roche Biochemicals) according to the manufacturer’s instructions. The cover slips were mounted on microscope glass slides using vectashield hard set mounting medium with DAPI (DAKO). A Zeiss Observer Z1 Microscope and Carl Zeiss LSM700 confocal microscope (Zeiss) were used for analysis. Images and confocal z-stacks were visualized with Zen 2012 software.

### Time-lapse experiments

Time-lapse experiments were performed at 37 °C in humidified 5 % CO_2_ in air, using a Nikon Biostation IM Live Cell Recorder (Nikon). The cells were cultured at a concentration of 1.5 × 10^5^ cells/ml, in time-lapse culture dishes (VWR) and pictures were taken every 10^th^ minute for up to 5 days.

### Transmission electron microscopy

#### Cells

Cells were briefly washed in PBS prior to fixation in 2.5 % glutaraldehyde (1 h or O/N in 4 °C). The dishes containing the cells were rinsed in 0.15 M Na-caccodylate and incubated for 1 h in 1 % osmium tetroxide in Na-caccodylate at RT followed by Na-caccodylate rinse. Dehydration was performed with 70 % ethanol for 30 min, 95 % ethanol for 30 min and 99.7 % ethanol for 1 h. A thin layer of newly made plastic (Agar 100 resin kit, Agar Scientific Ltd) was added for 1 h to permit evaporation of the alcohol. The plastic was poured off and a second plastic layer was added and left O/N in a desiccator. Next, plastic was heated up in an oven to enable its removal and a thicker, newly made plastic layer was added. The dishes were incubated in desiccator for 1–4 h before polymerization in oven (60 °C) for 48 h. Cells were sectioned using Leica ultracut UTC ultrotome (Rowaco AB) and studied in a Tecnai G2 transmission electron microscope (FEI Company). ORIUS SC200 CCD was used as camera and Gatan Digital Micrograph as software (both Gatan Inc.).

#### Microvesicles

Microvesicles were isolated by ultracentrifugation as described above and reconstituted in PBS. Samples were added onto a formvar-coated 200-mesh grid (Oxford Instruments) and incubated for 45 min in RT. The grid was dried and 1 % uranyl acetate was added for 10 s. Before analysis in a Tecnai G2 transmission electron microscope (FEI Company), the grid was dried for at least 15 min. ORIUS SC200 CCD was used as camera and Gatan Digital Micrograph as software (both Gatan Inc.).

### Cell lysates

For Aβ quantification studies, co-cultures of neurons and glia were seeded out in a concentration of 2.4 × 10^5^ cells/ml in 60 mm *x* 15 mm cell culture dishes (Corning). Following removal of the cell culture medium, 100 μl ice cold lysis buffer (20 mM Tris pH 7.5, 0.5 % Triton X-100, 0.5 % deoxycholic acid, 150 mM NaCl, 10 mM EDTA, 30 mM NaPyroPand protease inhibitor, Roche) was added to the dish. The lysed cells were collected using a cell lifter (Costar), transferred to Eppendorf tubes, incubated on ice for 30 min and centrifuged (30 min, 4 °C, 12 000 *x g*). The supernatant was collected and the remaining pellet was dissolved in 50 % formic acid and sonicated in 4 × 4 s pulses for 30 effective seconds at 50 % amplitude. Both the supernatants and pellets were stored in −70 °C until time of analysis by ELISA.

### Aβ_1-x_ and Aβ_x-42_ ELISAs

For Aβ_1-x_ ELISA [82] ninety-six well EIA/RIA plates (Corning Inc.) were coated O/N at 4 °C with the N-terminus (epitope 1-5) antibody mAb82E1 (100 ng/well, IBL-Hamburg) in PBS. Plates were blocked with 1 % bovine serum albumin (BSA) in PBS for 2 h in RT. Standard series of synthetic Aβ_42_ monomers (American Peptide) and samples (all except pellets of the lysates) were denatured by boiling for 5 min in 0.5 % sodium dodecyl sulfate (SDS) to avoid impaired detection caused by aggregated Aβ [83]. Before addition to plates, all SDS treated samples were diluted *x* 10 to decrease the SDS concentration and incubation followed for 2 h. Biotinylated mAb4G8 (0.3 μg/ml, Covance), specific for the mid region of Aβ, was used as secondary antibody and incubated for 1 h followed by incubation with streptavidin coupled HRP (1:2000, Mabtech AB) for 1 h. K-blue enhanced (Neogen Corporation) was used as HRP substrate and the reaction was stopped with 1 M H_2_SO_4_. Plates were measured by Tecan Infinite M200 PRO spectrophotometer (Tecan Group Ltd.) at 450 nm and analyzed with Magellan v7.0 software (Tecan Group Ltd.). Washing was performed by adding 250 μl washing buffer (phosphate buffered NaCl with 0.1 % Tween 20 and 0.15 % Kathon) *x* 3 repetitions between each step of the ELISA. All dilutions occurred in ELISA incubation buffer (0.05 % Tween, 0.1 % BSA and 0.15 % Kathon in PBS at pH 7.4). For the Aβ_x-42_ ELISA, polyclonal Aβ_42_ antibody (100 ng/well, Agrisera) and biotinylated mAb4G8 (0.5 μg/ml) was used as primary and secondary antibody, respectively. Aβ_x-42_ ELISA [13] was performed according to the same protocol as the Aβ_1-x_ ELISA, except for prolonged incubation times for blocking, sample (both O/N, 4 °C) and secondary antibody (2 h, RT) and increased SA-HRP dilution (1:5000). The pellet samples were neutralized with 1 M Trizma base + 0.5 M Na_2_HPO_4_ prior to the ELISA analysis.

### Aβ protofibril ELISA

Aβ protofibril ELISA using mAb158, was performed according to the protocol described by Englund et al. [41]. In short, ninety-six well EIA/RIA plates (Corning Inc.) were coated with 200 ng/well of mAb158 in 100 μl PBS at 4 °C O/N. Plates were blocked with 1 % BSA in PBS with 0.15 % Kathon. Standard series of synthetic Aβ_42_ protofibrils and samples were added to the plates for 2 h incubation in RT. All dilutions occurred in ELISA incubation buffer. After washing the plate, 0.5 μg/ml of biotinylated mAb158 was added and incubated for 1 h in RT. Subsequent steps and washing steps were performed as previously described in the *Aβ*_*1-x*_*ELISA* method.

### Immunostaining of mouse brain tissue

Twelve-fourteen-month-old tg-ArcSwe mice were perfused with isotonic saline solution followed by 4 % phosphate-buffered formaldehyde (Histolab AB). The brains were frozen and cryo-sectioned sagittally to a thickness of 14 μm and then permeabilized and blocked in 0.3 % Triton X-100 in PBS containing 5 % NGS for 1 h in RT. The sections were incubated at 4 °C O/N with primary antibody diluted in 0.3 % Triton/PBS with 0.5 % NGS and thoroughly washed in 0.3 % Triton/PBS. Thereafter, sections were incubated for 1 h in RT with secondary antibody diluted in 0.3 % Triton/PBS with 0.5 % NGS before repeating the washing step. The sections were mounted on microscope glass slides with Vectashield containing DAPI (Vector). Primary antibodies used were: rabbit anti-GFAP (1:400, DakoCytomation), mouse anti-GFAP (1:500, Sigma), rat anti-LAMP-2 (1:400, Abcam) and mAb158 (5 μg/ml). Secondary antibodies used were AlexaFluor 488 against mouse, rabbit and rat or AlexaFluor 555 against mouse and rabbit (1:500, Molecular probes).

### Western blot analysis

Microvesicle lysates were prepared as described above. 26 μl of each sample was loaded to a 4–12 % Bis-Tris Gel (NuPAGE, Life Technologies). Novex® Sharp Standard (Life technologies) was used as a standard. The gel was run for 1 h at 175 V in MES buffer (NuPage, Life Technologies) followed by transfer for 1 h at 20 V onto a PVDF membrane (Invitrogen). The membrane was blocked with 5 % BSA in TBS with 0.2 % Tween (TBS-T) for 1 h (RT), followed by washes in TBS-T and incubation with Flotillin-1 (mouse monoclonal, 1:500, BD Biosciences) antibody in 0.5 % BSA in TBS-T over night (4 °C). After extensive washes in TBS-T the membrane was incubated with a peroxidase-conjugated goat anti-mouse IgG (1:20 000, Pierce) antibody in 0.5 % BSA in TBS-T for 1 h (RT) and then finally washed again in TBS-T. The enhanced chemiluminescence (ECL) system (GE Healthcare) was used for development.

### Aβ inclusions, area, intensity measures and cell counting

Thirty images (10 images/cover slip from three independent cultures) were captured with an *x*40 objective on a Zeiss Observer Z1, using exactly the same settings. For the co-localization studies the images were manually analyzed. The cells were divided into four categories; cells with no detectable levels of Aβ_42_-555 protofibrils (−), cells with only small amounts of Aβ_42_-555 protofibrils (+), cells containing medium amounts (a couple of small Aβ_42_-555 protofibril inclusions or larger Aβ_42_ protofibril inclusions with low intensity of the 555-staining) (++) and cells with one or more large Aβ_42_-555 protofibril inclusions (+++). For Aβ intensity and area measurements, the images were analyzed with the Zen 2012 software (Zeiss). All area and intensity measurements of Aβ were set manually in the software and the number of viable cells was determined by manually counting cell nuclei in each field. Due to low cell numbers, the *x*20 objective was used when counting the number of viable neurons and oligodendrocytes. All images were analyzed in a blinded fashion.

### Statistics

All experiments were performed in triplicates with independent cell cultures derived from embryos of different pregnant mice. The results are presented in scatter plots or box plots with mean +/− standard deviation. Since the data was found not to meet the assumption of normal distribution, using the Shapiro-Wilk’s *W-*test, unpaired *t*-test and Kruskal-Wallis ANOVA, followed by Mann–Whitney *U*-test for pair-wise comparisons was used. Level of significance were set to *P < 0.05, ** < 0.01 and *** < 0.001.

## Additional files

Additional file 1: Aβ_42_ protofibril deposits are found in astrocytes, but not in neurons. Co-cultures stained for astrocytes (GFAP), neurons (βIII-tubulin) and Aβ_42_-555 protofibrils demonstrate that astrocytes, but not neurons, contain large deposits of Aβ_42_-555. Scale bars: 20 μm. (TIF 2925 kb) Additional file 2: Aβ inclusions co-localize with condensed nuclei of dead cells. Aβ_42_-555 protofibril exposed cultures labelled with TUNEL confirm that Aβ_42_-555 co-localizes with nuclei of dead cells. Scale bar: 20 μm. (TIF 716 kb) Additional file 3: Intracellular localization of large Aβ_42_-555 inclusions in astrocytes. Intersections from a 3D z-stack of an astrocyte exposed to Aβ_42_-555 protofibrils demonstrate that Aβ_42_-555 deposits are located intracellularly (arrow heads). DAPI (blue), GFAP (green) and Aβ_42_-555 (red). Scale bars: 10 μm. (TIF 2239 kb) Additional file 4: Immunostainings with Aβ_42_ specific antibodies confirm Aβ_42_ protofibril inclusions in astrocytes. After 24 h Aβ protofibril exposure, co-cultures were fixed and stained with GFAP and four different Aβ antibodies; mAb1C3 (a), 6E10 (b), mAb158 (c) and polyclonal Aβ_42_antibody (d). All antibodies detect extensive intracellular Aβ accumulation in astrocytes. Scale bars: 20 μm. (TIF 3705 kb) Additional file 5: Astrocytes differentiated in the presence of CNTF contain Aβ deposits. Pure (CNTF-treated) astrocytic cultures exposed to Aβ_42_-555 protofibrils demonstrate that astrocytes contain large intracellular deposits of Aβ_42_-555. Scale bar: 20 μm. (TIF 1446 kb) Additional file 6: Time-lapse movie from 30 min to 24 h following Aβ_42_-555 protofibril administration shows that astrocytes, but not neurons accumulate Aβ_42_-555 protofibrils over time. Images are captured in 10 min intervals. (MP4 5271 kb) Additional file 7: Aβ deposits in GFAP-positive astrocytes in the AD mouse brain. Immunohistochemistry of tg-ArcSwe mouse brain sections confirms that Aβ co-localizes with GFAP-positive astrocytes surrounding the plaques. Aβ (red), GFAP (green). Scale bar: 20 μm. (TIF 1591 kb) Additional file 8: No accumulation of Aβ_42_ monomers in the co-cultures. After 24 h Aβ_40_-555 monomer exposure of the cell cultures, no detectable Aβ_40_-555 is present in astrocytes (GFAP). Scale bar: 20 μm. (TIF 1768 kb) Additional file 9: Time-lapse movie starting 24 h after Aβ_42_-555 protofibril addition. Aβ_42_-555 accumulation induced formation of enlarged, dynamic vacuoles in the astrocytes. The giant vacuoles eventually shrink or collapse, concurrently as new vacuoles appear in the same astrocyte. Images are captured in 10 min intervals. (MP4 3765 kb) Additional file 10: Time-lapse movie captured at day 5 after Aβ_42_ protofibril addition. Enlarged vacuoles form and fuse in the astrocytes resulting in giant vacuoles with a diameter of ~50 μm. Images are captured in 5 min intervals. (MP4 2072 kb) Additional file 11: Aβ co-localizes with endosomal markers. Double stainings of Aβ_42_ protofibril exposed cell cultures with antibodies to Aβ/Rab5 or Aβ/Rab7 demonstrate a clear co-localization of Aβ inclusions and the early endosomal marker Rab5. Some co-localization is also noted for Aβ and the late endosomal marker Rab7. Scale bars: 20 μm. (TIF 1930 kb) Additional file 12: Aβ_42_ protofibrils do not directly induce neuronal cell death. Cortical neurons were exposed to 0.1 μM Aβ_42_ protofibrils for 48 h and TUNEL assay was performed to measure the number of apoptotic neurons. No significant differences are seen in the number of TUNEL positive cell nuclei or in the numbers of total cell nuclei compared to untreated neurons. (TIF 140 kb) Additional file 13: Aβ_42_ protofibril characterization. No considerable difference is noticed comparing serially diluted Aβ_42_ protofibrils and Aβ_42_-555 protofibrils with the Aβ protofibril selective mAb158 ELISA. (TIF 149 kb)

## Abbreviations

12 DIV: 12 days in vitro
AD: Alzheimer’s disease
Aβ: amyloid beta
BSA: bovine serum albumin
CNPase: 2',3'-Cyclic-nucleotide 3'-phosphodiesterase
CNTF: cilliary neurotrophic factor
CSF: cerebrospinal fluid
E14: embryonal day 14
ECL: enhanced chemiluminescence
GFAP: glial fibrillary acidic protein
HBSS: Hank’s buffered salt solution
LAMP-1: lysosome-associated membrane protein-1
LAMP-2: lysosome-associated membrane protein-2
MSD: multiple sulfatase deficiency
NGS: normal goat serum
PBS: phosphate buffered saline
SDS: sodium dodecyl sulfate
SEC: size-exclusion chromatography
TFEB: transcription factor EB
TUNEL: terminal (TdT)-mediated dUTP-biotin reaction mixture
