# Supplementary figures and images for: Accumulation of amyloid-β by astrocytes result in enlarged endosomes and microvesicle-induced apoptosis of neurons
Source: Mol Neurodegener. 2016 May 12;11:38. doi: 10.1186/s13024-016-0098-z (PMC4865996; doi:10.1186/s13024-016-0098-z)

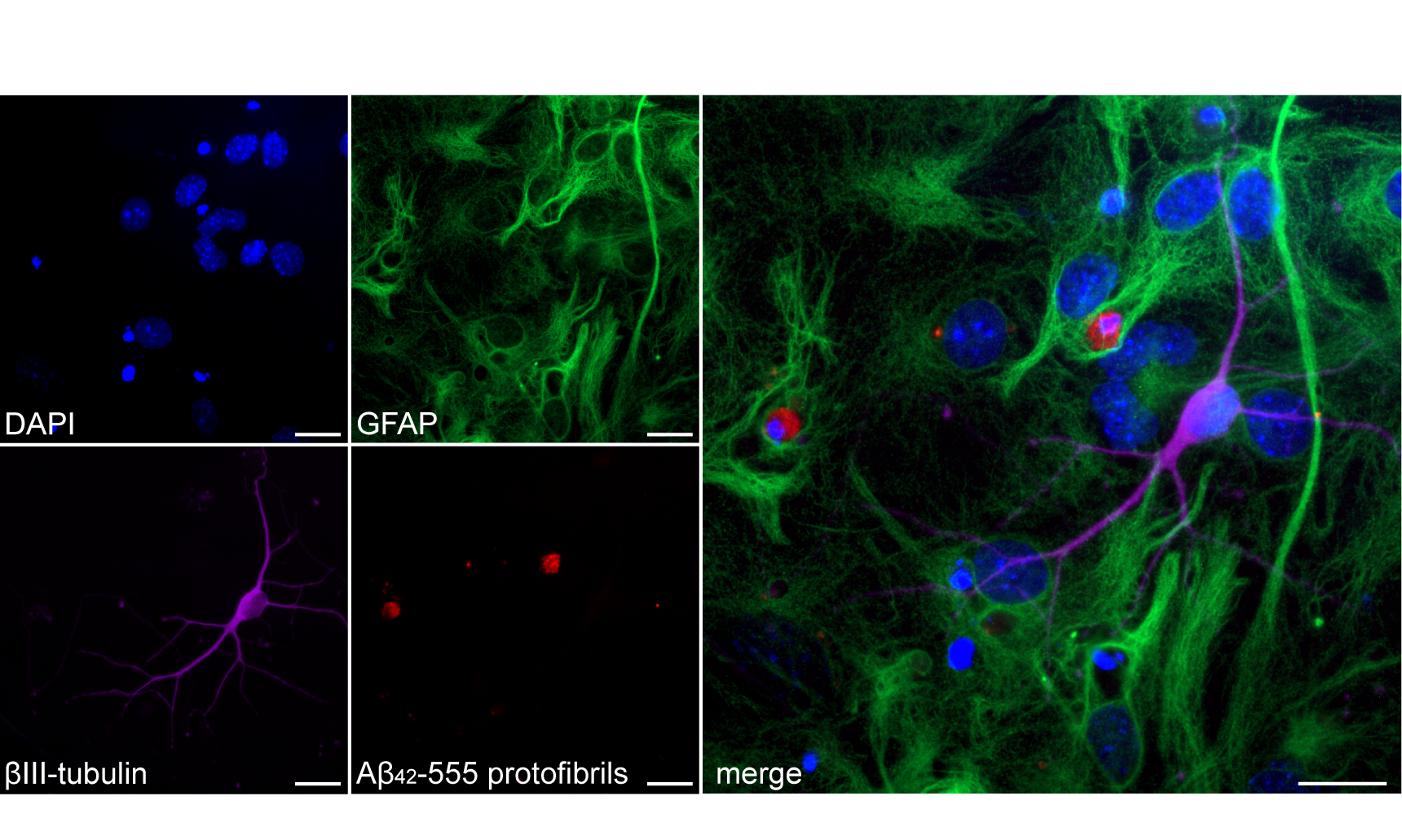

Supplement: Additional file 1: — Aβ42 protofibril deposits are found in astrocytes, but not in neurons. Co-cultures stained for astrocytes (GFAP), neurons (βIII-tubulin) and Aβ42-555 protofibrils demonstrate that astrocytes, but not neurons, contain large deposits of Aβ42-555. Scale bars: 20 μm. (TIF 2925 kb) [file 13024_2016_98_MOESM1_ESM.tif]

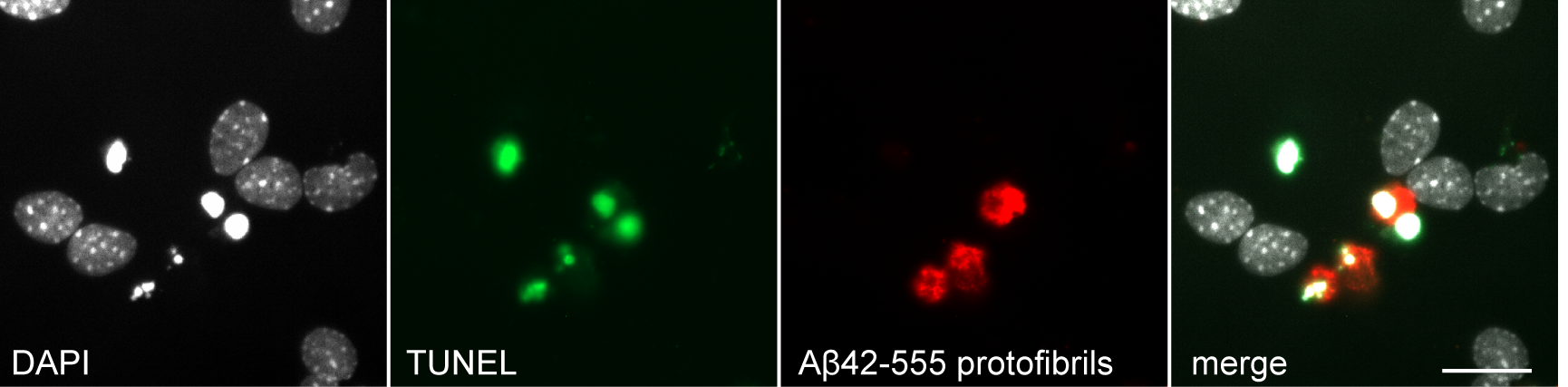

Supplement: Additional file 2: — Aβ inclusions co-localize with condensed nuclei of dead cells. Aβ42-555 protofibril exposed cultures labelled with TUNEL confirm that Aβ42-555 co-localizes with nuclei of dead cells. Scale bar: 20 μm. (TIF 716 kb) [file 13024_2016_98_MOESM2_ESM.tif]

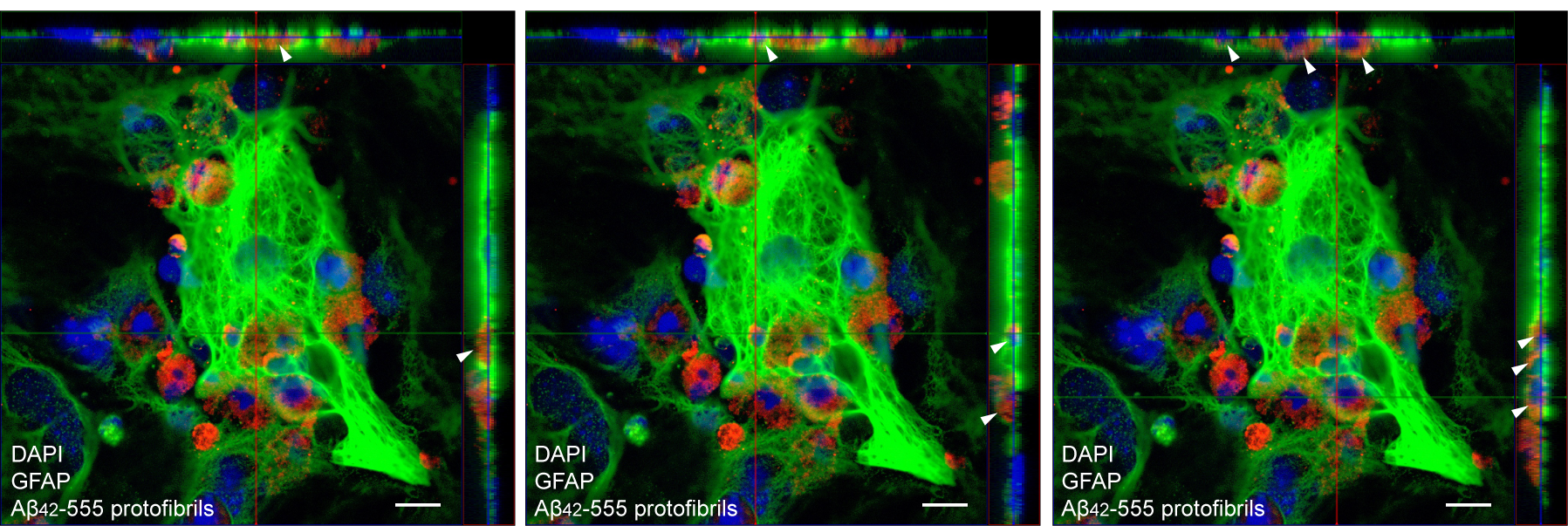

Supplement: Additional file 3: — Intracellular localization of large Aβ42-555 inclusions in astrocytes. Intersections from a 3D z-stack of an astrocyte exposed to Aβ42-555 protofibrils demonstrate that Aβ42-555 deposits are located intracellularly (arrow heads). DAPI (blue), GFAP (green) and Aβ42-555 (red). Scale bars: 10 μm. (TIF 2239 kb) [file 13024_2016_98_MOESM3_ESM.tif]

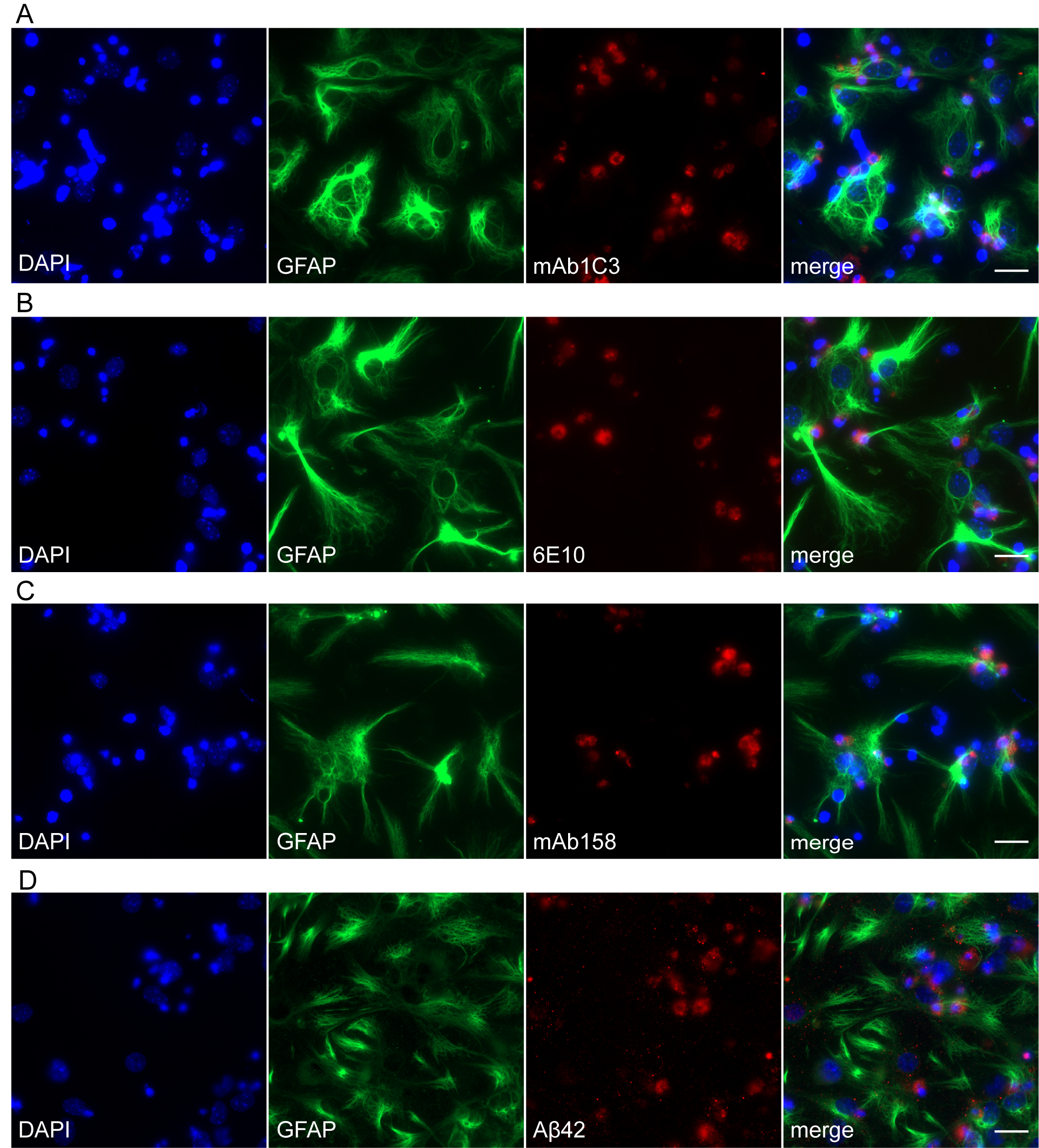

Supplement: Additional file 4: — Immunostainings with Aβ42 specific antibodies confirm Aβ42 protofibril inclusions in astrocytes. After 24 h Aβ protofibril exposure, co-cultures were fixed and stained with GFAP and four different Aβ antibodies; mAb1C3 (a), 6E10 (b), mAb158 (c) and polyclonal Aβ42antibody (d). All antibodies detect extensive intracellular Aβ accumulation in astrocytes. Scale bars: 20 μm. (TIF 3705 kb) [file 13024_2016_98_MOESM4_ESM.tif]

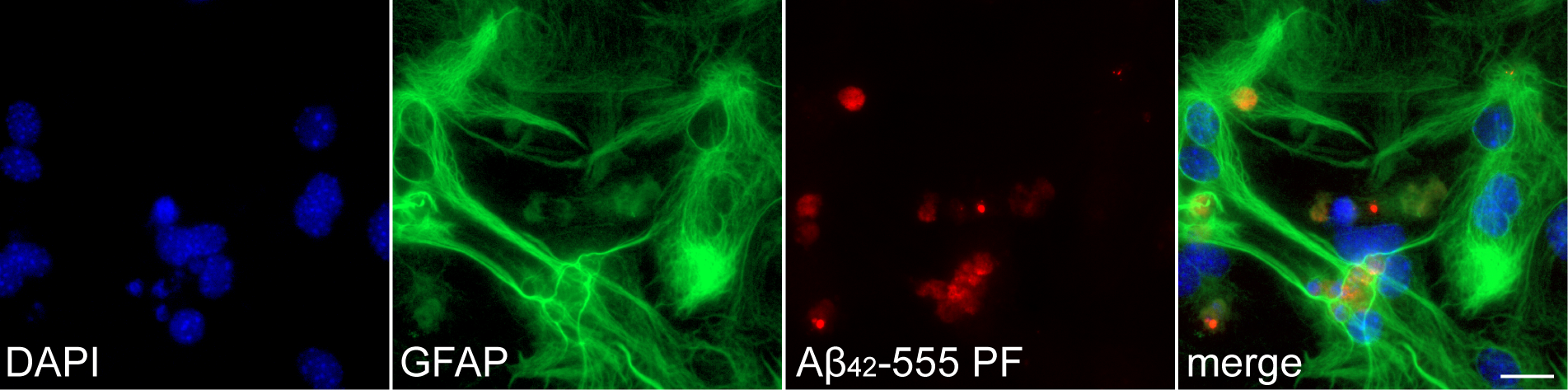

Supplement: Additional file 5: — Astrocytes differentiated in the presence of CNTF contain Aβ deposits. Pure (CNTF-treated) astrocytic cultures exposed to Aβ42-555 protofibrils demonstrate that astrocytes contain large intracellular deposits of Aβ42-555. Scale bar: 20 μm. (TIF 1446 kb) [file 13024_2016_98_MOESM5_ESM.tif]

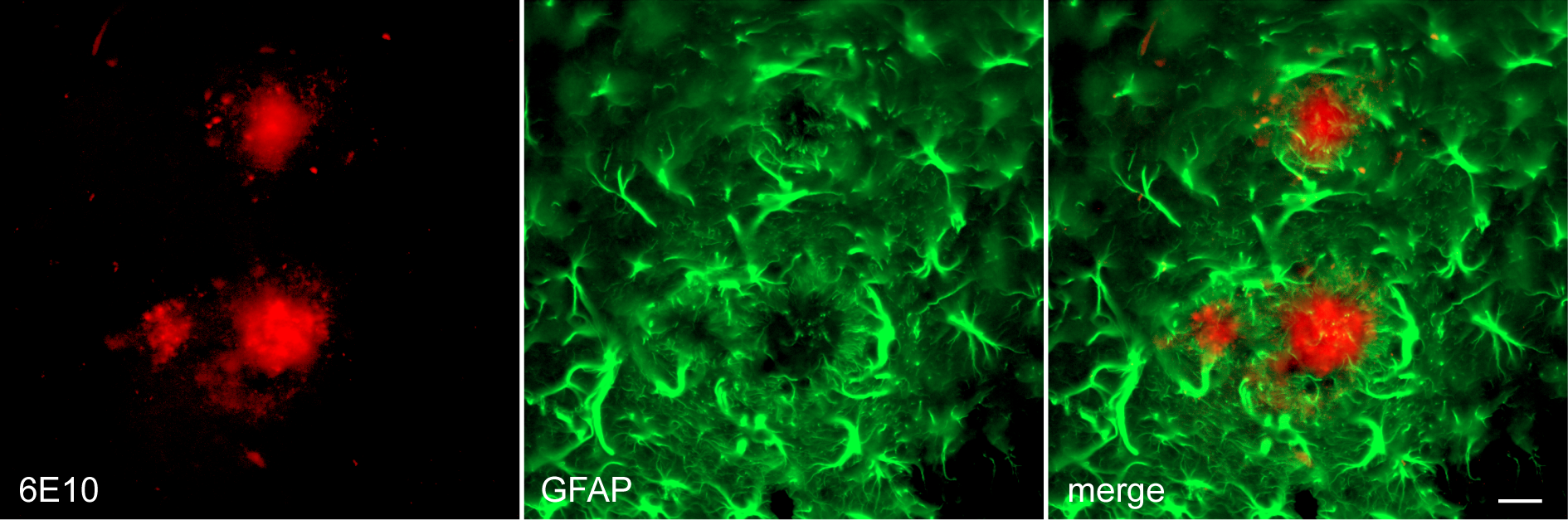

Supplement: Additional file 7: — Aβ deposits in GFAP-positive astrocytes in the AD mouse brain. Immunohistochemistry of tg-ArcSwe mouse brain sections confirms that Aβ co-localizes with GFAP-positive astrocytes surrounding the plaques. Aβ (red), GFAP (green). Scale bar: 20 μm. (TIF 1591 kb) [file 13024_2016_98_MOESM7_ESM.tif]

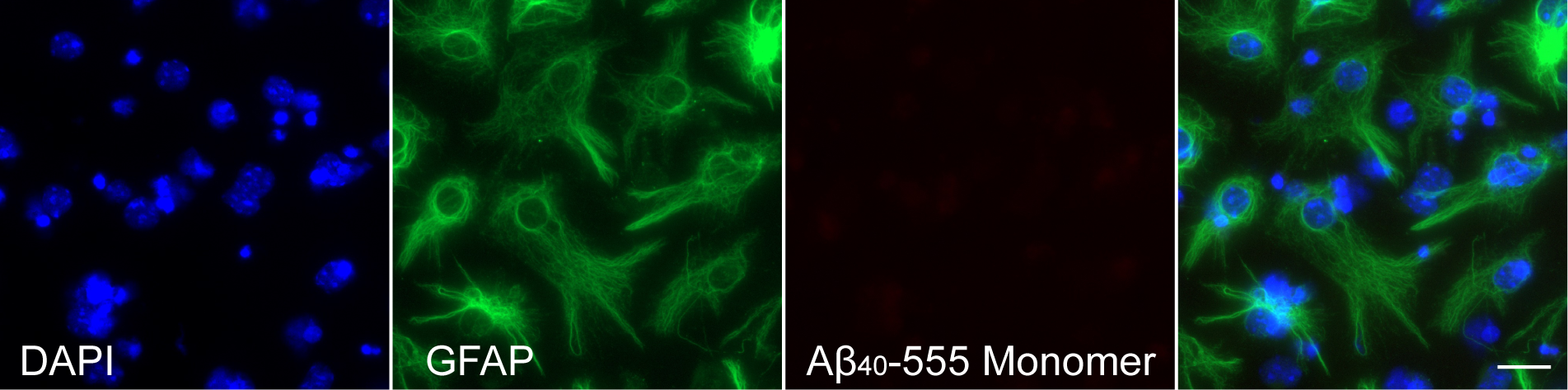

Supplement: Additional file 8: — No accumulation of Aβ42 monomers in the co-cultures. After 24 h Aβ40-555 monomer exposure of the cell cultures, no detectable Aβ40-555 is present in astrocytes (GFAP). Scale bar: 20 μm. (TIF 1768 kb) [file 13024_2016_98_MOESM8_ESM.tif]

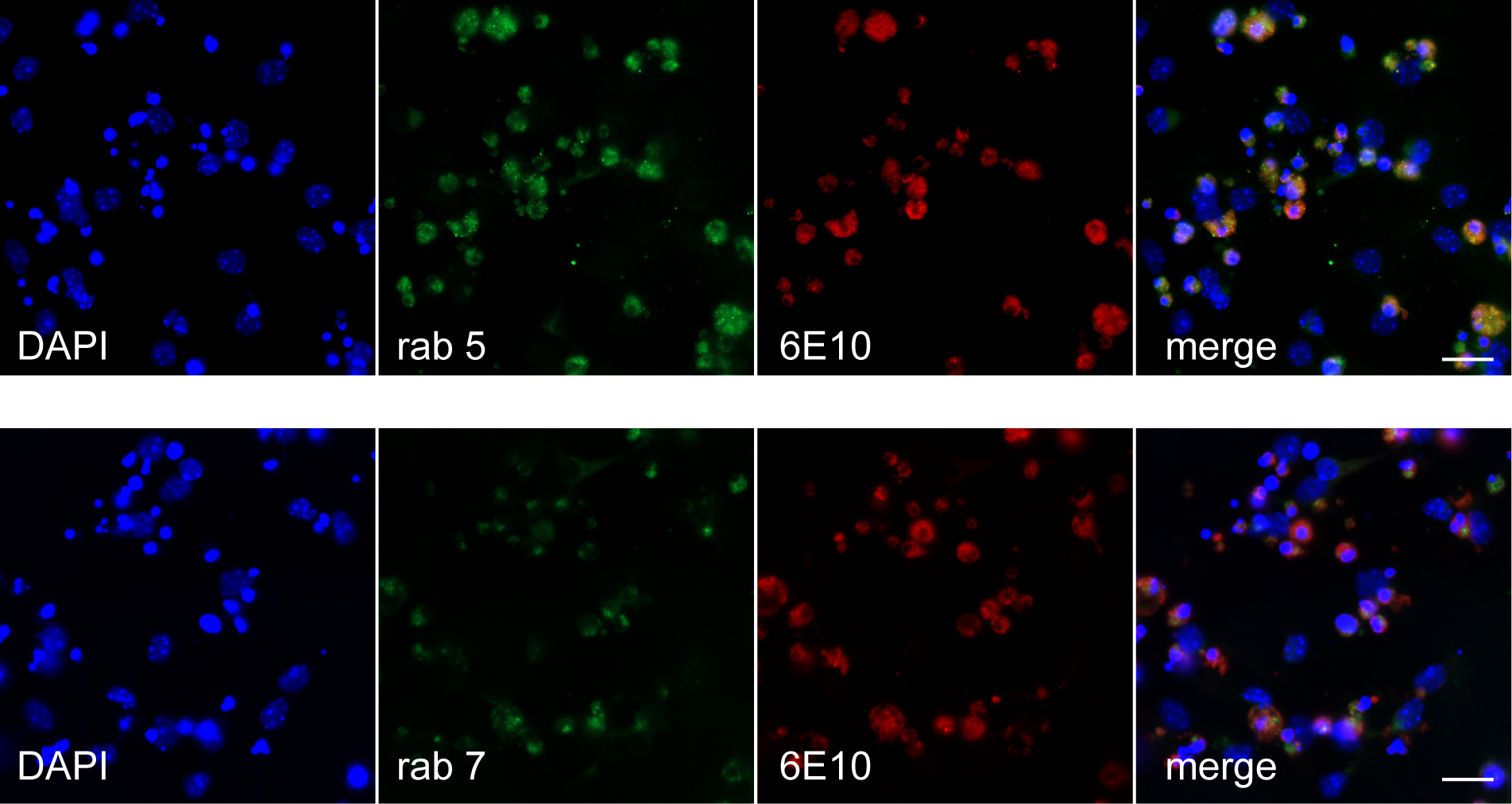

Supplement: Additional file 11: — Aβ co-localizes with endosomal markers. Double stainings of Aβ42 protofibril exposed cell cultures with antibodies to Aβ/Rab5 or Aβ/Rab7 demonstrate a clear co-localization of Aβ inclusions and the early endosomal marker Rab5. Some co-localization is also noted for Aβ and the late endosomal marker Rab7. Scale bars: 20 μm. (TIF 1930 kb) [file 13024_2016_98_MOESM11_ESM.tif]

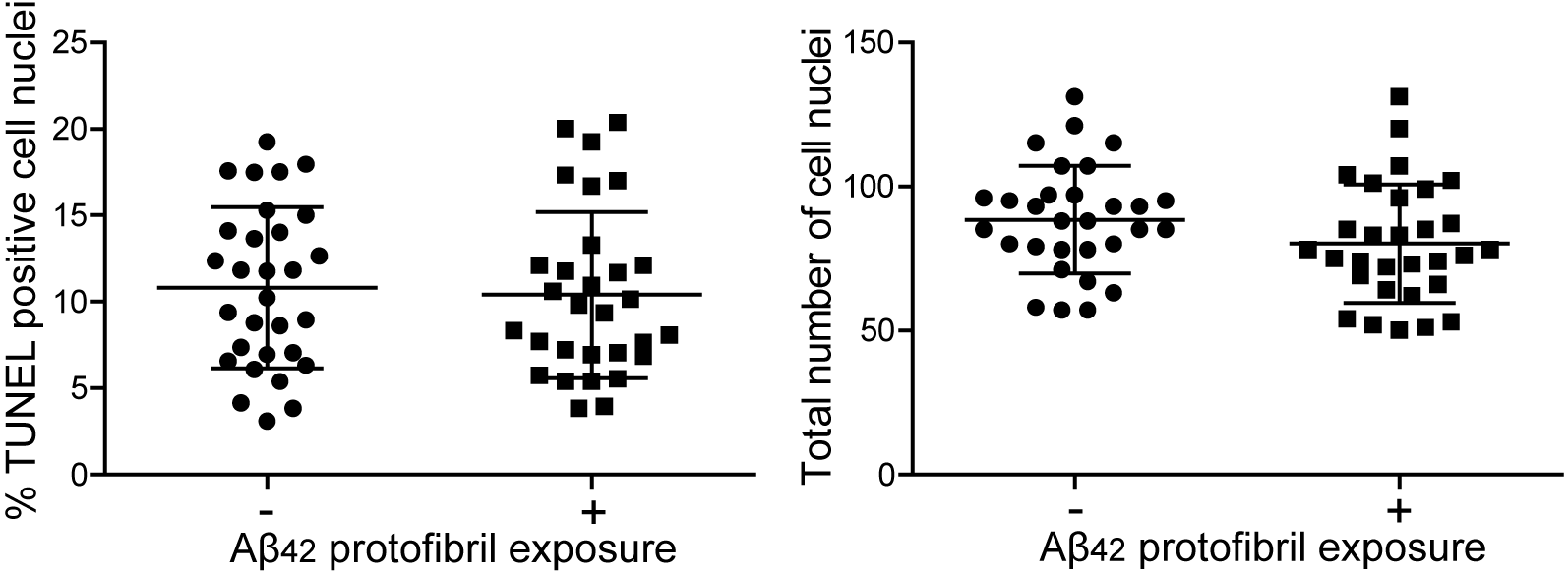

Supplement: Additional file 12: — Aβ42 protofibrils do not directly induce neuronal cell death. Cortical neurons were exposed to 0.1 μM Aβ42 protofibrils for 48 h and TUNEL assay was performed to measure the number of apoptotic neurons. No significant differences are seen in the number of TUNEL positive cell nuclei or in the numbers of total cell nuclei compared to untreated neurons. (TIF 140 kb) [file 13024_2016_98_MOESM12_ESM.tif]

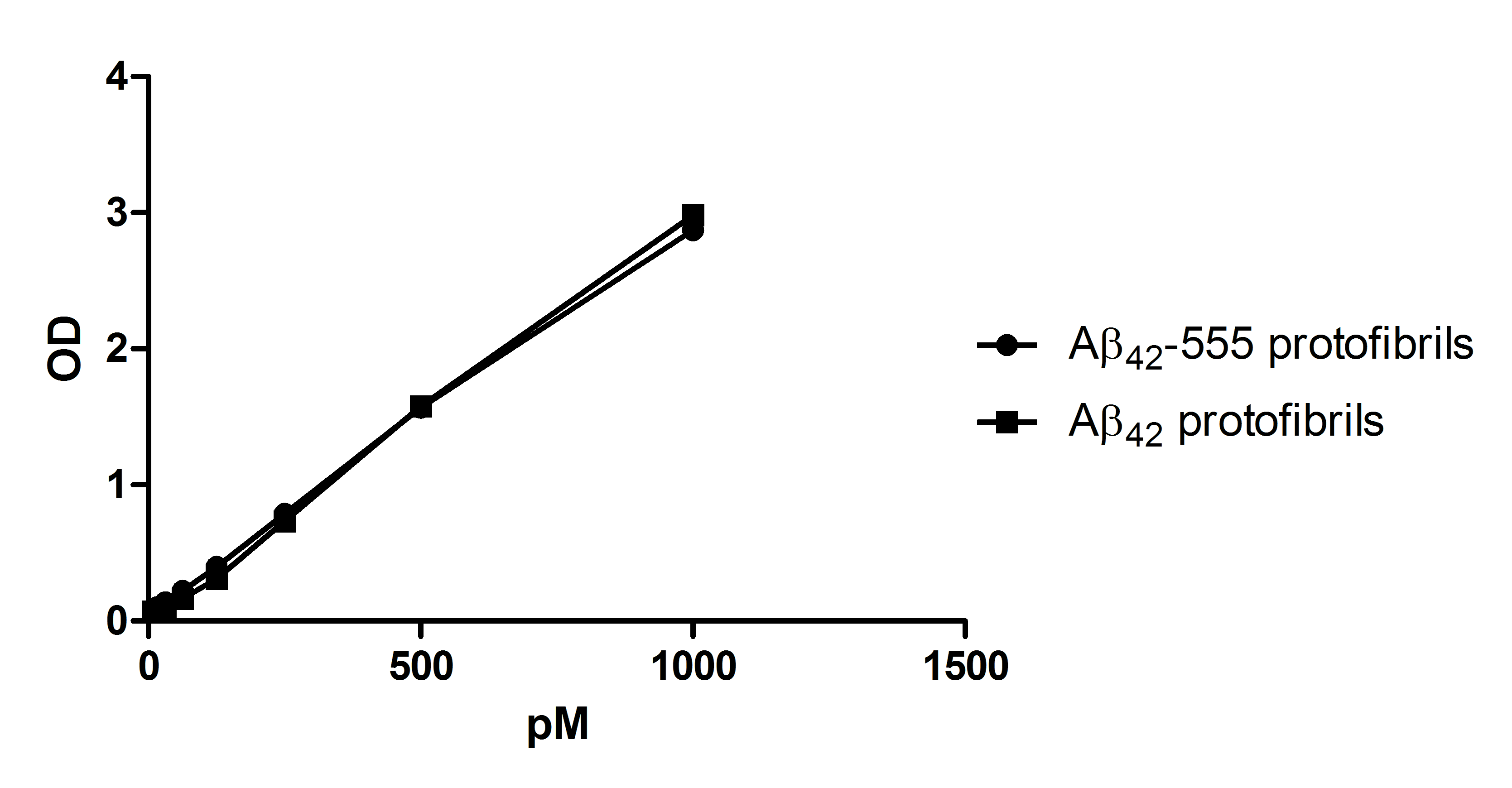

Supplement: Additional file 13: — Aβ42 protofibril characterization. No considerable difference is noticed comparing serially diluted Aβ42 protofibrils and Aβ42-555 protofibrils with the Aβ protofibril selective mAb158 ELISA. (TIF 149 kb) [file 13024_2016_98_MOESM13_ESM.tif]
